# Effect exerted by a hospital-home transition plan for people with Heart Failure: a randomized clinical trial^*^

**DOI:** 10.1590/1518-8345.8271.4942

**Published:** 2026-11-13

**Authors:** Claudia Marcela Vallejo-Ramírez, John Edison Cárdenas-Galvis, Gloria Carvajal-Carrascal, Alejandra Fuentes-Ramírez, Beatriz Sánchez-Herrera

**Affiliations:** 1 Universidad de Caldas, Facultad de Ciencias para la Salud, Manizales, Caldas, Colombia.; 2 Universidad de La Sabana, Facultad de Enfermería y Rehabilitación, Chía, Cundinamarca, Colombia.

**Keywords:** Nursing, Hospital to Home Transition, Patient Discharge, Heart Failure, Health Education, Nursing Theory

## Abstract

**Objective:**

to determine the effect exerted by *Plan Cuidarte* on care competence in people with heart failure during the hospital-home transition period.

**Method:**

a randomized clinical trial of the pre- and post-test that included 100 participants (Experimental Group=50, Comparison Group=50). The Experimental Group was applied *Plan Cuidarte*, which includes two stages: the first one consists in assessing discharge training and the second one deals with discharge training by means of educational processes. The Comparison Group was offered the conventional discharge plan. An intention to treat analysis was performed with intra- and inter-group comparisons and the dependent variable was *care competence*, measured with the *Cuidar* instrument. The data were analyzed by means of descriptive and analytical statistics, following all research ethics principles.

**Results:**

the Experimental group participants presented a statistically significant improvement in their care competence when compared to the Comparison Group, with a large effect size.

**Conclusion:**

*Plan Cuidarte* improves care competence in people with heart failure during the hospital-home transition. Based on the Theory of Transitions, this intervention has potential to be implemented in the discharge training aimed at this population segment. The trial protocol was registered in February 2024 under the following title: *Effect of the Plan Cuidarte on the Caregiving Competence of People With Heart Failure*, ID number NCT06309875 available at: https://clinicaltrials.gov/study/NCT06309875.

## Highlights

**(1)** The impact of Hansen’s disease and its influence on a person’s self-care.

**(2)** *Plan Cuidarte* exerted a large effect on care competence.

**(3)** The intervention was effective in the hospital-home transition for HF.

**(4)** Nurses’ role is fundamental in early hospital discharge.

**(5)** *Plan Cuidarte* is based on applied Nursing theory.

## Introduction

Cardiovascular diseases (CVDs) are the most frequent cause of mortality at the global level, with higher incidence in low- and middle-income countries^1^. Heart Failure (HF) is but one of the CVD manifestations: approximately 1 out of 4 individuals will develop HF at some moment in their life^2^. In Latin America, HF presents high burden of the disease with significant prevalence and mortality, and is considered a Public Health problem with 1% prevalence in this region^3^. It is also important to consider that patients with HF present high hospital readmission rates: in a study conducted in Brazil, the percentage of hospital readmissions among people with HF was 26.2% at 90 days, with an in-hospital mortality rate of 10.9%, and 27.7% accumulated incidence of mortality at 12 months^4^.

The prevalence of HF in Colombia is 2.3%, affecting approximately 1,097,201 people^5^. At the national level, the hospitalization costs almost double the ones for outpatient care^6^. Patients with HF are frequently exposed to hospital admissions and readmissions; consequently, they are continuously faced with hospital-home transition processes and it has been documented that they face challenges such as medication use, harmful habits like smoking and drinking alcohol, exposure to stressful situations in these processes; in addition, deficient professional follow-up during hospital-home transitions has been reported^7^.

In that sense, it is necessary to implement strategies that improve the care for people with HF, which due to its chronic disease nature, requires those who suffer it to be competent enough for self-care. A number of studies conducted in Colombia have reported that people with chronic diseases lack optimum care competence^8^. Adequate care competence levels are fundamental in order to preserve safety during this transition process^7^, as it is a vulnerable moment for people with HF that is characterized by high readmission and in-hospital mortality rates. This period demands comprehensive patient-centered training offered before discharge^9^.

Given the current trend to reduce hospitalization times to a minimum, the risks are higher mainly during the period encompassing the first three months after a hospitalization due to HF^6^; in addition to its personal, social and economic costs, its chronicity requires implementing specific interventions for this population group that may improve their care competence.

The hospital-home transition is a Nursing phenomenon originating from Meleis’ Theory of Transitions; it is a systemic, vulnerable and multidimensional process and it is necessary to design complex interventions to address it. Hospital-home transitions have been designated as transitional care: they are critical periods, especially for patients with HF, who require careful management to prevent readmissions and improve their evolution^10^.

This research integrates three concepts from the Theory of Transitions: Conditions for the Transition; Nursing Therapeutics and Response Patterns^11^. The Conditions for the transition exert an influence on achieving a healthy transition (care goal according to this theory). It is fundamental to consider them in the design of customized interventions, as it has been shown that individualized discharge training adapted to specific needs improves outcomes in patients with HF^12^. The development of Nursing interventions targeted at this transition training is framed under the concept of Nursing Therapeutics.

It has been recommended that patient education interventions on aspects such as diet, physical activity and stress management (among others) should be included as part of the non-pharmacological treatment^13^.

It is for this reason that Nursing has sought various mechanisms to support care for people with HF, improve their health status and avoid complications by means of suitable planning for the hospital-home transition. A review of the Nursing interventions to provide care to people with HF during this transition discovered that Nursing education and follow-up favor independence in care and reduce the number of readmissions and the mortality rates. However, the authors report that these interventions are not frequently used, that the educational content fails to reach the specialization level required and that they are not standardized^14^. Another study, which reviewed multi-component programs with educational and follow-up interventions, detected that they reduced mortality, readmission and hospitalization days^6^. Reports about discharge plans targeted at patients with HF seeking to enhance their care competence are added to the above^15^; however, and despite these advances, the evidence is still weak.

In addition to designing Nursing interventions that cater for the hospital-home transition, it is fundamental to assess and socialize their impact. Given the aforementioned, one of the subconcepts from the theory that is used in the one of outcome indicator, which allows determining if the result of a given transition is healthy or not, where each individual shows mastery of the skills and behaviors required to handle their new situations. It is based on these indicators that patient-centered health outcomes are achieved, such as satisfaction and care skills^10^.

Given the high incidence and the personal, social and economic costs inherent to the people with HF that are treated at three institutions form the Caldas department, Colombia, it was necessary to structure a plan for the hospital-home transition that was specific to them.

Given the above, the following hypothesis was set forth for the current study: the *Plan Cuidarte* intervention (based on the theoretical concept of Nursing Therapeutics) exerts a positive effect on care competence, with an increase of at least 5 points (Subconcept: Outcome indicators), among the people with HF undergoing the hospital-home transition at three hospitals from Manizales (when compared to conventional discharge care), where *Plan Cuidarte* is an acronym corresponding to Early and Structured Care Plan (*Plan de Cuidado Anticipado y Estructurado*) for the hospital-home transitional care of patients with HF.

The objective of the current study was to determine the effect exerted by *Plan Cuidarte* on care competence in people with HF during the hospital-home transition period.

## Method

### Study design

A parallel Randomized Controlled Trial (RCT) of superiority, with Comparison Group and pre-/post-intervention measuring instances; it included a previous pilot test that allowed adjusting the initial protocol It was not possible to blind the participants due to the educational nature of the intervention; however, the information analysts were in fact blinded by providing them with de-identified and coded data.

In the intervention preparation and design phase, both the patients and the general target audience supported the design by reviewing and validating the educational material and its pedagogical delivery modality, which contributed to better understanding and accepting it. The institutions reviewed the protocol and supported its qualification.

### Data collection site and period

The recruitment and follow-up activities lasted from March to August 2024 and were performed at three health institutions from the city of Manizales, Caldas, Colombia.

### Population

Individuals over 18 years old hospitalized with HF diagnoses.

### Selection criteria

The individuals included were those over the age of 18 years old, admitted to the hospitalization service with HF diagnoses at 3 hospital centers and with no mental or functional alterations that precluded their care autonomy. The HF diagnoses were determined by reviewing the medical charts and by confirming the information with the health personnel in charge of each patient. Both cognitive state and functionality level were assessed based on components from the Characterization survey for the care of the caregiver-person with chronic disease dyad (GCPC-UN-D)^16^, namely: the Short Portable Mental Status Questionnaire (SPMSQ - PFEFFER) and the PULSES scale, acronym for “stability of the Pathology”, “Use of upper limbs”, “Locomotion”, “Sensory capacity”, “Elimination” and “Social activity”. The individuals excluded were those that were candidates for cardiac transplants in the next 6 months and those who were transferred to intensive or intermediate care units. The hospital centers included should comprehensively assist people with HF.

### Definition of the sample

Sampling was of the non-probability type and for convenience. The sample size was calculated considering Type I and Type II errors at 0.05, effect size (Cohen’s d) at 0.8 and 95% power, with a known attrition estimated at 20% according to what was reported in the previous pilot study. The initial sample calculation suggested 100 participants in the sample, distributed between the Experimental and Comparison groups with 50 subjects each. 1:1 random allocation was applied both to the Experimental and to the Comparison groups; the randomization units were the study participants and the process was in charge of a third person, supported by a computer generator using permutation randomization. The research assistants (who allocated the participants to the groups) were unaware of the sequence as they could only know the allocation group via their mobile phones at the allocation time.

### Study variables

The primary outcome evaluated was the care competence level, the study dependent variable, defined as follows: a patient’s capacity, skill and preparedness to perform care actions. It encompasses six dimensions: Knowledge (Notions about the disease), Uniqueness (Tools to face challenges), Instrumental (Skills to perform care actions), Enjoyment (Personal well-being and satisfaction degree), Anticipation (Notions to anticipate to future needs) and Social relations and interactions (Social ties and support networks)^17^. Satisfaction with the intervention was evaluated as secondary outcome. Contextual variables such as knowledge, access and use of Information and Communication technologies (ICTs) in care, perception of burden and support in care were measured as independent variables, in addition to defining the participants’ demographic characterization.

### Instruments used and data collection

The initial characterization in the first phase of *Plan Cuidarte* was implemented by means of the GCPC-UN-D survey, validated in Colombia^16^; from it, the questions targeted at the patient were applied, thus determining the demographic characterization, the perception about burden and support, and ICT use.

The participants’ care competence was measures using the *Cuidar* instrument (short patient version) validated in Colombia, which reached a Cronbach’s Alpha value of 0.928^17^. *Cuidar* includes the six care competence dimensions, which are assessed by means of 20 questions scored with a Likert scale. Care competence can be categorized as high (49-60), average (37-48) or low (0-36) and was measured before initiating the intervention or usual care (T0) and after the intervention or usual care (T1); the mean values of the dimensions and the overall care competence were employed for the analysis and comparisons.

Finally, the satisfaction level with the intervention targeted at hospital discharge was assessed, measured after the intervention in the Experimental Group of after the usual care in the Comparison Group (T1) with a survey developed *ad hoc* for the current study, reason why it still lacks validation studies.

### Intervention and comparison

The Experimental Group was applied *Plan Cuidarte* for people with HF. It is a Nursing intervention targeted at assisting hospital-home transitions in this population segment. *Plan Cuidarte* met the parameters for developing systematic interventions proposed by Whittemore^18^. In order to ensure reliability in development of the protocol, an intervention manual was designed where the sequence to implement the intervention, how to apply and interpret the instruments and a checklist to assess reliability were specified, thus enabling the intervention to be applied as planned.

The intervention design was conceptually guided by the Theory of Transitions^19^, as described below.

### Applying the theory to design the intervention

This study resorted to Afaf Ibrahim Meleis’ middle-range Theory of Transitions as framework to design the intervention proposed: *Plan Cuidarte*, as described below.

*Nature of the transition*: the hospital-home transition is theoretically classified as a health and disease transition. For people with HF, it has been described under the term of “transitional care”^10^.

*Nursing Therapeutics*: measures that can be applied to therapeutic interventions during transitions; above all, the interventions are grounded on evaluation and education processes^10^. Considering the vulnerability implied by transitional care in patients with HF, the *Plan Cuidarte* intervention is designed, targeted at enhancing the patients’ care competence during their hospital-home transition.

The *Plan Cuidarte* intervention consists of two stages underpinned by two theoretical subconcepts:

*Stage 1. Evaluation regarding preparedness for the hospìtal-home transition*: it aims for patients with HF to enjoy full knowledge about their preparedness for the transition by being aware of the *Conditions for the transition* (theoretical subconcept). In order to evaluate these conditions, the questions corresponding to the patient assessment from the GCPC-UN-D survey were applied, which assess aspects related to contextual variables that can exert an influence on the hospital-home transition process; the *Cuidar* instrument (patient version) was also used in this stage to assess the baseline care competence (pre-test). This assessment process was conducted once for each patient in 30-40 minutes.

*Stage 2. Hospital-home transition training:* after assessing preparedness for the transition and the contextual variables, the training was initiated with educational processes. The *Plan Cuidarte* intervention has a solid educational component arranged in 2 sessions: the first one addressed the *Knowledge* dimension (1 session lasting 20-30 minutes), whereas the second one dealt with the *Instrumental* and *Anticipation* dimensions (1 session lasting 30-40 minutes), which are *care competence* dimensions. The guiding questions were applied at the end of each educational sessions to assess the participants’ understanding about the educational material, following the Teach Back (TB) Method guidelines to identify and bridge gaps or errors in the information. This method has shown to improve multiple outcomes in patients with HF^20^. A booklet and a digital didactic module were employed in this phase; the educational sessions included a definition of HF, follow-up of signs and symptoms, adherence to pharmacological and non-pharmacological treatments, identification of warning signs and decision-making according to the national and international recommendations for HF care.

*Response Patterns:* this concept is used to determine if the outcome of a given transition is healthy or not after implementing the intervention. *Plan Cuidarte* was evaluated by means of the following response indicators: patients’ satisfaction with the training intervention for the hospital-home transition; and care competence after the intervention (post-test).

The intervention was developed in-person and in an individualized way during the hospitalization period; the participants from both groups continued to be offered the corresponding clinical care concomitantly. The intervention was applied by research assistants that were Nursing students duly trained in this process.

In turn, the Comparison Group was offered the usual discharge assistance, which consisted in providing the general care indications on the discharge date. The participants were explained the discharge recommendations, including the administrative process to request control appointments, procedures and medications. In addition, a charge nurse explained the necessary care measures for using the medications prescribed.

### Data treatment and analysis

These processes were conducted by intention to treat and by protocol. The data were stored in Excel sheets and analyzed in SPSS, version 19. The sociodemographic data were analyzed by means of descriptive statistics and the Chi-square test was employed for the comparisons. In the “care competence” variable, the intra-group comparison was developed with Wilcoxon’s test; in turn, Mann-Whitney’s U test was used for the inter-group comparison. The effect size analysis was performed with Cohen’s *d* and, given the sample size, an adjustment was made using Hedges’ *g*. The patients’ satisfaction with the intervention received was handled with a percentage analysis.

### Ethical considerations

The study was conducted according to national and international ethical guidelines and was approved by the Ethics Committee of *Universidad de Caldas* on February 24^th^, 2023, file code CBCS-004. It was authorized by the participating health institutions. Each participant was advised about the study purpose and details, also obtaining their informed consent.

As for identification of harms, a non-systematic assessment was made, considering that this research is of minimum risk. A preliminary analysis was performed, considering the potential risk that some questions from the instruments might trigger feelings of anxiety, sadness, fear or uncertainty, among others. Consequently, this aspect was included in the informed consent form specifying to the patients the possibility of spontaneously expressing such sensations, of being provided the necessary assistance and of withdrawing from the study in case they wished so.

## Results

The study report followed the Consolidated Standards of Reporting Trials (CONSORT)^21^ for reporting randomized clinical trials. A total of 150 participants were assessed for eligibility: 42 failed to meet the inclusion criteria and 8 refused to participate, thus achieving a 92% recruitment rate. Consequently, 100 participants were randomized (50 to each group) and analyzed by intention to treat. Likewise, a secondary analysis was performed by protocol with a sample comprised by 46 individuals from the Experimental group and 37 from the Comparison Group (participants that finished the study), with 98% sample efficiency. The follow-up loss rate was 17% (Figure 1).

**Figure 1:**
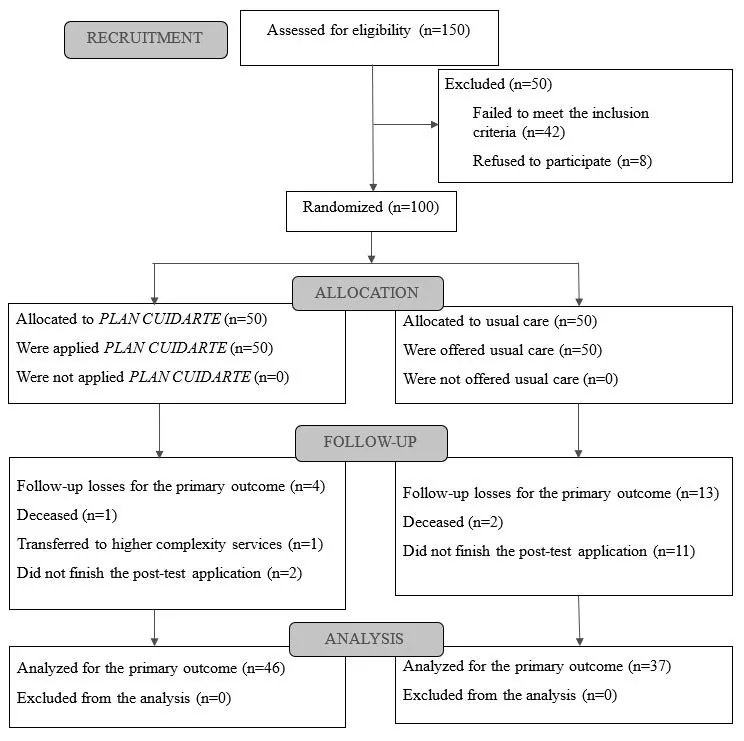
Flowchart^21^ describing the participants

### Demographic characteristics

The participants’ mean age was 70±10.8 and 69.8±11.1 for the Experimental and Comparison groups, respectively (*p*=0.199) (Mann-Whitney’s U). In all, 48% of the Experimental Group patients and 60% from the Comparison Group lived in the capital city of the Caldas Department, Colombia. The participants’ minimum and maximum time since their HF diagnoses were 1 month and 30 years, respectively. No statistically significant differences (*p*>0.05) were found in the comparison made between the demographic characteristics presented in each group, which indicated the baseline comparability of the groups for these variables (Table 1).

**Table 1:** Sociodemographic variables corresponding to the patients from the Experimental and Comparison groups (n = 100). Colombia, 2024

Variables	Categories	Experimental Group		Comparison Group		p*
		n=50		n=50		
		Frequency	Percentage	Frequency	Percentage	
Gender	Male	30	60	32	64	0.680*
	Female	20	40	18	36	
Schooling	No studies	5	10	2	4	0.711*
	Incomplete Elementary School	20	40	19	38	
	Complete Elementary School	6	12	5	10	
	Incomplete High School	9	18	11	22	
	Complete High School	5	10	9	18	
	Technician/Technologist	1	2	2	4	
	Complete Higher Education	4	8	2	4	
Marital status	Single	12	24	10	20	0.560*
	Married	18	36	25	50	
	Separated/Divorced	4	8	2	4	
	Widowed	10	20	10	20	
	Consensual union	6	12	3	6	
Occupation	House chores	29	58	30	60	0.787*
	Employee	3	6	5	10	
	Autonomous worker	13	13	12	24	
	Other	5	5	3	6	
Socioeconomic strata	1 (Low)	18	36	12	24	0.140*
	2	17	34	16	32	
	3	6	12	17	34	
	4	4	8	2	4	
	5	1	2	0	0	
	6 (High)	4	8	3	6	
Has only one caregiver	Yes	36	72	43	86	0.140^†^
	No	14	28	7	14	

*p-value obtained in the Chi-square test; ^†^ *p*-value obtained in Fisher’s exact test

### Burden and support perceptions, and ICT use

As for the perception about care burden and support, the satisfaction level with the latter was slightly higher in the Comparison Group in all the dimensions evaluated (psychological, family, religious, economic and social support); however, no statistically significant differences were found between the groups, which ratified their baseline comparability regarding these variables: psychological support (*p*=0.201), family support (*p*=0.085), religious support (*p*=0.194), economic support (*p*=0.234) and social support (*p*=0.412).

As for employing ICTs in care, the Experimental Group presented limited knowledge about, access to and use of computers and Internet, with more than 62% at low levels. Cell phones achieved better results, with knowledge, access and use levels between average and high, but without full leverage. Traditional media such as TV and radio presented high knowledge and access levels (nearly 56%). A similar pattern was noticed in the Comparison Group, but slightly more favorable; nevertheless, low levels also predominated in computers and Internet (70% in knowledge and 72% in access). Cell phones showed better leverage, with nearly 50% at high access and knowledge levels and predominantly average use. The limited leverage of computers and Internet was repeated in both groups, when compared to better leverage of cell phones and high TV and radio use levels.

### Intra-group care competence

In the intra-group pre-test and post-test comparisons there were changes in the Experimental group, indicating a significant improvement (*p*=0.000) in care competence, which rose from the low to the average level; care competence remained at the low level in the Comparison Group (Table 2).

**Table 2:** Intra-group comparisons between the participants from the Experimental and Comparison groups (n = 100). Colombia, 2024

Care competence dimensions	Experimental Group (n=50)						
	Pre-test*			Post-test^†^			p^‡^
	Mean	SD^§^	Level	Mean	SD^§^	Level	
Knowledge	1.84	2.034	Low	6.26	2.761	Average	0.000
Uniqueness	8.4	1.884	Average	8.54	3.138	Average	0.036
Instrumental	2.1	2.261	Low	5.56	2.442	Average	0.000
Enjoying well-being conditions	7.38	2.089	Average	7.3	3.303	Average	0.811
Anticipation	1.12	1.48	Low	3.56	1.875	Average	0.000
Support network and relations	9.04	2.515	Average	9.62	3.458	Average	0.134
Overall care competence	29.88	8.178	Low	40.84	14.148	Average	0.000
**Care competence dimensions**	**Comparison Group (n=50)**						
	Pre-test*			**Post-test^†^**			**p^‡^**
	**Mean**	**SD^§^**	**Level**	**Mean**	**SD^§^**	**Level**	
Knowledge	1.94	1.921	Low	2.4	2.109	Low	0.154
Uniqueness	8.74	2.174	Average	6.5	4.464	Low	0.009
Instrumental	2.22	1.765	Low	2.6	2.312	Low	0.277
Enjoying well-being conditions	6.9	1.876	Average	5.74	4.309	Average	0.074
Anticipation	1.22	1.282	Low	1.66	1.547	Low	0.122
Support network and relations	9.62	2.506	Average	7.28	4.656	Average	0.005
Overall care competence	30.64	6.03	Low	26.18	16.999	Low	0.697

*Before the intervention; ^†^After the intervention; ^‡^ *p*-value obtained in Wilcoxon’s test; ^§^Standard deviation

### Inter-group care competence

In relation to care competence, there were statistically significant differences between the Comparison and Experimental groups at the end of the intervention. The Experimental Group patients presented better care competence in the knowledge, instrumental, anticipation (*p*=0.000), uniqueness (*p*=0.036) and relations (*p*=0.005) dimensions and in the overall care competence (*p*=0.000); as for the enjoying well-being conditions dimensions, there was an improvement in the Experimental Group, although it was not statistically significant (Table 3).

**Table 3:** Inter-group comparisons between the participants from the Experimental and Comparison groups (n = 100). Colombia, 2024

Care competence dimensions	Experimental Group (n=50)		Comparison Group (n=50)		p^†^
	Mean	SD*	Mean	SD*	
Knowledge	6.26	2.761	2.4	2.109	0.000
Uniqueness	8.54	3.138	6.5	4.464	0.036
Instrumental	5.56	2.442	2.6	2.312	0.000
Enjoying well-being conditions	7.3	3.303	5.74	4.309	0.076
Anticipation	3.56	1.875	1.66	1.547	0.000
Support network and relations	9.62	3.458	7.28	4.656	0.005
Overall care competence	40.84	14.148	26.18	16.999	0.000

*Standard Deviation; ^†^ *p*-value obtained in Mann-Whitney’s U test

A large effect size was observed in favor of the Experimental Group (Cohen’s *d* =0.94; Hedges’ *g*=0.93; 95%CI: 0.52-1.35), which indicates a clinically relevant difference between both groups in the outcome analyzed in the comparison between the Experimental Group (Mean=40.84, Standard Deviation=14.14, sample size=50) and the Comparison Group (Mean=26.18, Standard Deviation=16.99, sample size=50). This result indicates a substantial difference between the groups, with a large effect size that suggests practical relevance in the variable analyzed^22^ ^-^ ^23^.

Likewise, a data analysis by protocol was performed including the participants that finished the intervention; a large size effect was observed (Cohen’s *d* =1.19 with 95% confidence interval) in the comparison between the Experimental Group (Mean=44.39, Standard Deviation=7.54, sample size=46) and the Comparison Group (Mean=35.38, Standard Deviation=7.67, sample size=37). This result indicates that the intervention exerted a positive effect.

Considering the sample size of the current study, an adjustment was made calculating Hedges’ *g*, as it provides an unbiased estimate of the effect size, especially suitable for finite sample sizes. The *g* value obtained was 1.18 [95% Confidence Interval (0.71;1.64)], which indicates that the intervention exerted a large effect on care competence.

### Satisfaction with the hospital-home transition training intervention

Most of the participants from the Experimental Group rated the *Plan Cuidarte* intervention as excellent, underscoring the quality of the educational content offered for HF care, the tools used and the attitude shown by the personnel that taught it (74%, 80% and 76% of the participants rated these characteristics as excellent, respectively). As for the Comparison Group, most of the participants rated the discharge training process as good, highlighting the need to improve aspects such as using didactic tools.

## Discussion

This study assessed the effect exerted by the *Plan Cuidarte* Nursing intervention implemented during the hospital-home transition on the care competence of people with HF, comparing it to conventional discharge care. The individuals that were offered *Plan Cuidarte* presented better outcomes than the Comparison Group (with statistically significant differences) in the care competence dimensions related to knowledge, instrumentation and anticipation, as well as in the overall care competence level.

The literature points out that Nursing interventions based on theories produce better results than conventional care^24^. In this study, the Theory of Transitions allowed structuring the *Plan Cuidarte* intervention, which outdid the conventional discharge plan. Likewise, the Whittemore framework^18^ eased designing and evaluating this structured intervention with replication potential to favor evidence-based practice.

Based on these findings and thanks to patient education as an aid to improve and control their disease, it is expected that the people that were offered *Plan Cuidarte* enjoy a fluid hospital-home transition since, as it has been documented, the above is crucial to improve outcomes in patients with HF^25^.

These findings ratify those from other studies at the national level conducted with patients suffering chronic diseases, which have reported that structured discharge plans improve such competence^8^.

This positive change achieved at the care competence level thanks to *Plan Cuidarte* responds to the need to strengthen this group of patients, among whom it has been reported that their care competence was not optimum to handle their health conditions before implementing a discharge program for people with cardiovascular diseases^8^.

The current research also ratifies findings about the epidemiological profile of patients with HF reported in previous studies. Both in this case and on the other documented ones, most of the patients diagnosed with HF are older adults and male^6^ ^,^ ^26^ ^-^ ^28^.

The findings on schooling, marital status, socioeconomic strata and having only one caregiver reported in the current research also coincide with previous studies^5^.

In addition, the results evidence an important gap in digital literacy, especially in terms of technologies that require advanced technical skills; this underscores the need to strengthen training in computer and Internet use. A study about the experience of patients with HF in ICT use for self-management reports that this low literacy level can trigger negative experiences, reason why it is recommended to provide professional support by incorporating new technologies to the care provided to them^29^. This support should be offered as a significant element to be included in the health care of these people, using ICTs in the transitional care of patients with HF can improve their quality of life and satisfaction with the assistance provided and even reduce the number of hospital readmissions^30^.

It is important to note that 3% of the hospitalized patients died during the current research. This coincides with what is reported in the *Recolfaca* study: 3%-10% of the patients hospitalized due to HF die in the hospital^5^. This high mortality evidences once again the need to develop interventions to prevent and control decompensation cases, as well as to reduce the number of hospital readmissions among patients with HF^6^.

As for the implications of this research for the practice and for future studies, it is essential to consider that HF is the main cause of hospitalization in people aged over 65 years old^31^. Therefore, it is pertinent to assess the effect exerted by *Plan Cuidarte* on clinical outcomes such as hospital readmissions, visits to emergency services and mortality. Consequently, a longitudinal approach is required to analyze the impact exerted by the interventions throughout time and in different population groups, especially older adults, who are more vulnerable to post-discharge complications^32^. As already proposed, this follow-up might incorporate Telehealth, Telemonitoring, phone follow-up or home visit tools^6^, which would imply integrating digital training as part of the care process.

Likewise, in future studies it will be important to analyze the effectiveness of *Plan Cuidarte* by including caregivers of patients with HF, as the interventions targeted at discharge have shown to improve care competence in patient/caregiver dyads^8^.

The results of the current study highlight the importance of continue developing and implementing structured and customized Nursing intervention programs that may improve care competence among patients with HF. These programs should be integrated to the hospital discharge process and ensure a fluid transition to outpatient care in a more frequent and continuous manner and led by Nursing professionals^33^.

Likewise, it is crucial to improve the patients’ care competence for them to manage their disease, seeking the best possible clinical results and a positive handling experience^34^. *Plan Cuidarte* can be useful, rigorous and comprehensive for patients with HF that need to extend their care at their homes.

Despite the findings obtained in this research, it is necessary to point out that the care competence reported by the patients with HF corresponds to a self-reported datum and that it was evaluated during a brief follow-up period. This limits the possibility of determining their practical implications and cost-benefit ratio in clinical and post-discharge outcomes such as quality of life, overall health condition, onset of complications, readmissions, hospitalization time or mortality rates, among others. Likewise, it is to be stated that these findings refer to a specific group of patients and that the study should be replicated so that they can be generalized to other population segments or contexts.

## Conclusion

*Plan Cuidarte* is a systematized and theoretically grounded intervention that, when applied during hospitalization, significantly improves care competence in people with HF in the hospital-home transition period.

Its methodological structure and the fact that is grounded on conceptual frameworks inherent to the discipline support its replicability and pertinence to integrate it to the usual clinical practice. The results show that the Nursing professionals’ active participation is essential to favor safer transitions, to contribute to better managing the disease and to attaining more favorable health outcomes. Consequently, the study reasserts the strategic value of Nursing interventions at hospital discharge and contributes diverse evidence that strengthen effective and sustainable practices in the care of people with chronic diseases such as HF.

## Data Availability

The dataset for this article is available at the following link https://figshare.com/account/articles/29361920?file=55938617
