# Supplementary material for: Effect exerted by a hospital-home transition plan for people with Heart Failure: a randomized clinical trial
Source: Rev Lat Am Enfermagem. 2026 Nov 13;34:e4942. doi: 10.1590/1518-8345.8271.4942 (PMC13762693; doi:10.1590/1518-8345.8271.4942)
Supplement: Translation

# Translation: Efeito de um plano de transição do hospital para casa em pessoas com Insuficiência Cardíaca: um ensaio clínico randomizado^*^

**DOI:** 10.1590/1518-8345.8271.4943

**Authors:** Claudia Marcela Vallejo-Ramírez, John Edison Cárdenas-Galvis, Gloria Carvajal-Carrascal, Alejandra Fuentes-Ramírez, Beatriz Sánchez-Herrera

**Affiliations:** 1 Universidad de Caldas, Facultad de Ciencias para la Salud, Manizales, Caldas, Colombia.; 2 Universidad de La Sabana, Facultad de Enfermería y Rehabilitación, Chía, Cundinamarca, Colombia.

**Keywords:** Enfermagem, Transição do Hospital para o Domicílio, Alta do Paciente, Insuficiência Cardíaca, Educação em Saúde, Teoria de Enfermagem.

## Abstract

**Objetivo:**

determinar o efeito do *Plan Cuidarte* na competência para o cuidado de pessoas com insuficiência cardíaca durante o período de transição do hospital para o domicílio.

**Método:**

ensaio clínico randomizado de tipo pré-teste/pós-teste com 100 participantes (Grupo Experimental=50, Grupo Comparação=50). O Grupo Experimental foi tratado com o *Plan Cuidarte,* que consiste em duas etapas: a primeira avalia a preparação para a alta hospitalar e a segunda foca na preparação para a alta por meio de processos educativos. O Grupo Comparação recebeu o plano de alta convencional. Foi realizada uma análise por intenção de tratar com comparações intra e intergrupos, sendo a variável dependente a *competência para o cuidado* mensurada pelo instrumento *Cuidar*. Os dados foram analisados utilizando estatística descritiva e analítica, seguindo os princípios éticos em pesquisa.

**Resultados:**

os participantes do Grupo Experimental apresentaram melhora estatisticamente significativa na competência para o cuidado em relação ao Grupo Comparação, com grande magnitude de efeito.

**Conclusão:**

o *Plan Cuidarte* melhora a competência para o cuidado de pessoas com insuficiência cardíaca durante a transição do hospital para o domicílio. Com base na teoria da transição, essa intervenção tem potencial para ser implementada na preparação para a alta hospitalar dessa população. Protocolo de ensaio clínico registrado em fevereiro de 2024, título do protocolo: *Effect of the Plan Cuidarte on the Caregiving Competence of People with Heart Failure*, número de identificação NCT06309875, disponível em: https://clinicaltrials.gov/study/NCT06309875.

## Destaques

**(1)** O Plan Cuidarte aprimora a competência no atendimento à insuficiência cardíaca.

**(2)** O Plan Cuidarte teve grande magnitude de efeito na competência para o cuidado.

**(3)** A intervenção foi eficaz na transição do hospital para o domicílio em pacientes com insuficiência cardíaca.

**(4)** O papel do enfermeiro é fundamental na alta hospitalar precoce.

**(5)** Compreensão das pessoas afetadas pela doença sobre autocuidado.

## Introdução

As doenças cardiovasculares (DCV) são a principal causa de morte em todo o mundo, com maior incidência em países de baixa e média renda^1^. Uma das manifestações das DCV é a insuficiência cardíaca (IC): aproximadamente 1 em cada 4 pessoas desenvolverá IC durante a vida^2^. A IC na América Latina apresenta uma alta carga de doença, com frequência e mortalidade elevadas, sendo considerada um problema de saúde pública, com prevalência de 1% nessa região^3^. Outrossim, é importante considerar que pacientes com IC apresentam altas taxas de reinternação hospitalar: em um estudo realizado no Brasil, pessoas com IC apresentaram uma taxa de reinternação hospitalar de 26,2% aos 90 dias, com uma taxa de mortalidade intra-hospitalar de 10,9% e uma incidência acumulada de mortalidade aos 12 meses de 27,7%^4^.

Na Colômbia, a prevalência de IC é de 2,3%, afetando aproximadamente 1.097.201 pessoas^5^. O custo da hospitalização a nível nacional representa quase o dobro do custo dos cuidados ambulatórios^6^. Os pacientes com IC estão frequentemente sujeitos a hospitalizações e reinternações hospitalares; em consequência, enfrentam continuamente o processo de transição do hospital para o domicílio e está documentado que, neste processo, afrontam desafios como as dificuldades na utilização da medicação, a presença de hábitos nocivos como o tabagismo e o consumo de álcool, a exposição a situações estressantes, sendo também relatado um acompanhamento profissional insuficiente durante a transição do hospital para o domicílio^7^.

Nesse sentido, é necessário implementar estratégias que melhorem o cuidado de pessoas com IC, que, devido à sua natureza crônica, exige que os portadores sejam competentes em autocuidado. Estudos realizados na Colômbia relataram que pessoas com doenças crônicas não possuem competência ideal para o autocuidado^8^. Níveis adequados de competência em cuidados são essenciais para manter a segurança durante esse processo de transição^7^, visto que este é um período vulnerável para pessoas com IC, caracterizado por altas taxas de reinternação e mortalidade hospitalar. Este período requer preparação integral e centrada no paciente, fornecida antes da alta^9^.

Dada a tendência atual de minimizar a duração da internação hospitalar, os riscos são maiores principalmente durante o período dos primeiros três meses após a hospitalização por IC^6^. Igualmente, o seu custo pessoal, social e econômico, aliado à cronicidade da condição, torna necessário implementar intervenções específicas para este grupo populacional que melhorem a sua competência para o cuidado.

A transição do hospital para o domicílio é um fenômeno de Enfermagem enraizado na Teoria das Transições de Meleis; trata-se de um processo sistêmico, vulnerável e multidimensional que requer intervenções complexas. A transição do hospital para o domicílio tem sido denominada como “transições de cuidado”: esses são períodos críticos, especialmente para a população com IC, que requer um manejo cuidadoso para prevenir reinternações e melhorar os resultados^10^.

Esta pesquisa integra três conceitos da Teoria das Transições: Condições para a Transição, Terapêutica de Enfermagem e Padrões de Resposta^11^. As Condições para a Transição influenciam a obtenção de uma transição saudável (o objetivo do cuidado segundo essa teoria). É essencial considerá-las no planejamento de intervenções personalizadas, visto que a preparação individualizada para a alta, adaptada às necessidades específicas, demonstrou melhorar os desfechos em pacientes com IC^12^. O desenvolvimento de intervenções de enfermagem voltadas para a preparação para a transição se enquadra no conceito de Terapêutica de Enfermagem.

Foi recomendado que, como parte do tratamento não farmacológico, as intervenções de educação do paciente sejam incluídas em aspectos como dieta, atividade física e gestão do estresse, entre outros^13^.

Por isso, a Enfermagem tem buscado diversos mecanismos para apoiar o cuidado de pessoas com IC, melhorar seu estado de saúde e evitar complicações por meio de um planejamento adequado para a transição do hospital para o domicílio. Uma revisão de intervenções de Enfermagem para o cuidado de pessoas com IC durante essa transição constatou que a educação e o acompanhamento em Enfermagem promovem a independência no cuidado e diminuem as reinternações e a mortalidade. No entanto, as autoras relatam que essas intervenções não são frequentemente utilizadas, que a educação carece do nível necessário de especialização e que não são padronizadas^14^. Outro estudo que revisou programas multicomponentes com intervenções de educação e acompanhamento constatou que estes reduziram a mortalidade, as reinternações e o tempo de internação^6^. Igualmente, foram relatados planos de alta direcionados a pacientes com IC buscando fortalecer suas competências para o cuidado^15^; no entanto, apesar desses avanços, as evidências ainda são fracas.

Além de planejar intervenções de Enfermagem para lidar com a transição do hospital para o domicílio, é essencial mensurar e compartilhar seu impacto. Portanto, um subconceito da teoria utilizada refere aos indicadores de resultados, visto que permitem determinar se o resultado de uma transição é saudável ou não. Nesses indicadores os indivíduos demonstram o domínio das habilidades e comportamentos necessários para lidar com suas novas situações. A partir deles é possível identificar os resultados de saúde centrados no paciente, tais como satisfação e a competência para o cuidado^10^.

Considerando a alta incidência e o custo pessoal, social e econômico das pessoas com IC atendidas em três instituições no departamento de Caldas, na Colômbia, tornou-se necessário estruturar um plano de cuidados para a transição do hospital ao domicílio, específico para esse grupo.

Portanto, para o presente estudo, foi proposta a seguinte hipótese de pesquisa: a intervenção *Plan Cuidarte* (Baseado no conceito teórico da Terapêutica de Enfermagem) modifica positivamente a competência para o cuidado, com um aumento de pelo menos 5 pontos (Subconceito: Indicador de Resultado), de pessoas com IC que estão na transição do hospital para o domicílio em três hospitais de Manizales, em comparação com o cuidado convencional na alta hospitalar, sendo *Plan Cuidarte* a sigla para Plano de Cuidado Antecipatório e Estruturado para o cuidado da transição do hospital para o domicílio de pacientes com IC.

O presente estudo teve como objetivo determinar o efeito do *Plan Cuidarte* na competência para o cuidado de pessoas com IC durante o período de transição do hospital para o domicílio.

## Método

### *Design* do estudo

Ensaio clínico randomizado (ECR) controlado paralelo, de superioridade, com Grupo Comparação e medições pré e pós-intervenção, conduzido com teste-piloto prévio para ajustar o protocolo inicial. O cegamento dos participantes não foi possível devido à natureza educacional da intervenção; contudo, manteve-se o cegamento dos analistas da informação por meio do fornecimento de dados anonimizados e codificados.

Durante a fase de preparação e concepção da intervenção, tanto os pacientes quanto o público em geral apoiaram o *design*, revisando e validando os materiais educativos e sua aplicação pedagógica, o que contribuiu para uma melhor compreensão e aceitação. As instituições revisaram o protocolo e apoiaram sua qualificação.

### Local e período de coleta de dados

Período de recrutamento e acompanhamento de março a agosto de 2024, em três instituições de saúde na cidade de Manizales, Caldas, Colômbia.

### População

Pessoas com mais de 18 anos de idade hospitalizadas com diagnóstico de insuficiência cardíaca (IC).

### Critérios de seleção

Foram incluídos indivíduos com mais de 18 anos de idade hospitalizados no serviço de internação com diagnóstico de insuficiência cardíaca (IC) em três centros hospitalares, desde que não apresentassem comprometimento mental ou funcional que impedisse a autonomia em seus cuidados pessoais. O diagnóstico de IC foi determinado por meio da revisão de prontuários médicos e confirmação com a equipe de saúde responsável pelo paciente. O estado cognitivo e o nível funcional foram avaliados utilizando dois componentes do Questionário de Caracterização para o Cuidado da Díade Cuidador-Pessoa com Doença Crônica (GCPC-UN-D)^16^: *Short Portable Mental Status Questionnaire* (SPMSQ - PFEFFER) e a escala PULSES, acrônimo que corresponde a *stability of the Pathology* (estabilidade da patologia), *Use of upper limbs* (uso de membros superiores), *Locomotion* (locomoção), *Sensory capacity* (capacidade sensorial), *Elimination* (eliminação) y *Social activity* (atividade social). Indivíduos que fossem candidatos a transplante cardíaco nos próximos seis meses ou que tivessem sido transferidos para cuidados intensivos ou intermediários foram excluídos. Os hospitais incluídos eram obrigados a fornecer atendimento integral a pessoas com IC.

### Definição da amostra

A amostragem foi não probabilística por conveniência. O tamanho da amostra foi calculado considerando os erros Tipo I (0,05) e Tipo II (0,05), com um poder estatístico de 95% e um tamanho de efeito de 0,8 (*d*de Cohen). Adicionalmente, previu-se uma taxa de atrito de 20%, conforme estimado no estudo-piloto. Anteriormente, o cálculo inicial do tamanho da amostra sugeriu 100 participantes, distribuídos entre os grupos Experimental e de Comparação, com 50 participantes em cada grupo. Uma alocação aleatória de 1:1 foi realizada para ambos os grupos. A unidade de randomização foram os participantes do estudo, e o processo foi realizado por uma terceira parte, com o auxílio de um gerador computacional que utilizava randomização por permutação. Os assistentes de pesquisa, responsáveis pela alocação dos participantes aos grupos, desconheciam a sequência, uma vez que só podiam saber o grupo ao qual tinham sido atribuídos por meio de seus telefones celulares no momento da alocação.

### Variáveis do estudo

O desfecho primário avaliado foi o nível de competência para o cuidado, a variável dependente do estudo, definida como a capacidade, habilidade e preparo do paciente para prestar cuidados. Compreende seis dimensões: Conhecimento (compreensão da doença); Singularidade (ferramentas para lidar com os desafios); Instrumental (habilidades para realizar as tarefas de cuidado); Satisfação (grau de bem-estar e satisfação pessoal), Antecipação (conhecimento para antecipar necessidades futuras) e Relacionamento e Interação Social (relações sociais e redes de apoio)^17^. O desfecho secundário avaliado foi a satisfação com a intervenção. As variáveis independentes incluíram fatores contextuais como conhecimento, acesso e uso de Tecnologias de Informação e Comunicação (TICs) para o cuidado, carga e apoio percebidos no cuidado e características demográficas.

### Instrumentos utilizados e coleta de dados

A caracterização inicial realizada na primeira fase do *Plan Cuidarte* realizou-se utilizando o instrumento GCPC-UN-D validado na Colômbia^16^. A partir dele, foram aplicadas as questões orientadas para o paciente com as quais foram determinadas a caracterização demográfica, a percepção de sobrecarga e apoio e o uso das TICs.

A competência para o cuidado foi medida utilizando o instrumento *Cuidar* (versão curta para pacientes), validado na Colômbia, cujo alfa de Cronbach foi de 0,928^17^. O instrumento inclui as seis dimensões da competência no cuidado, avaliadas por meio de 20 perguntas com escala Likert. A competência no cuidado pode ser categorizada como alta (49-60), média (37-48) ou baixa (0-36) e foi mensurada antes do início da intervenção ou do cuidado usual (T0) e após a intervenção ou o cuidado usual (T1); para análise e comparações, foi utilizada a média das dimensões e da competência geral no cuidado.

Por fim, avaliou-se a satisfação com a intervenção para alta hospitalar, medida após a intervenção no Grupo Experimental ou após o atendimento padrão no Grupo Comparação (T1), com um questionário desenvolvido *ad hoc* para o presente estudo, razão pela qual ainda não possui estudos de validação.

### Intervenção e comparação

O Grupo Experimental recebeu a intervenção *Plan Cuidarte* para pessoas com insuficiência cardíaca. Essa intervenção de Enfermagem foi concebida para apoiar a transição do hospital para o domicílio nessa população. O *Plan Cuidarte* seguiu os parâmetros de Whittemore para o desenvolvimento de intervenções sistemáticas^18^. Para garantir a adesão ao protocolo, foi elaborado um manual de intervenção especificando a sequência de implementação da intervenção, a aplicação e interpretação dos instrumentos e uma lista de verificação para avaliação da adesão, assegurando, assim, que a intervenção fosse administrada conforme planejado.

O desenho da intervenção foi conceitualmente guiado pela Teoria das Transições^19^, conforme detalhado a seguir.

### Aplicação da Teoria ao planejamento de intervenções

Este estudo adotou a Teoria de Médio Alcance das Transições de Afaf Ibrahim Meleis como estrutura para o desenvolvimento da intervenção proposta: *Plan Cuidarte*, conforme descrito abaixo:

*Natureza da transição*: a transição do hospital para o domicílio é teoricamente classificada como uma transição de saúde e doença. Para pessoas com IC, ela tem sido descrita sob o termo transição de cuidados^10^.

*Terapêutica de Enfermagem*: trata-se de medidas que podem ser aplicadas às intervenções terapêuticas durante as transições; notadamente, as intervenções baseiam-se em processos de avaliação e educação^10^. Considerando a vulnerabilidade envolvida nas transições de cuidados para pacientes com IC, foi concebida a intervenção *Plan Cuidarte*, que visa fortalecer a competência para o cuidado ao paciente durante a transição do hospital para o domicílio.

A intervenção *Plan Cuidarte* consiste em duas etapas guiadas por dois subconceitos teóricos:

*Etapa 1. Avaliação da preparação para a transição do hospital para o domicílio*: esta etapa visa gerar uma compreensão abrangente da preparação do paciente com IC para a transição, por meio do conhecimento das *condições para a transição* (subconceito teórico). Para avaliar essas condições, foram aplicadas as questões correspondentes à seção de avaliação do paciente do instrumento GCPC-UN-D, que avalia aspectos relacionados a variáveis contextuais que podem influenciar o processo de transição do hospital para o domicílio. Nesta etapa, o instrumento *Cuidar* (versão para pacientes) também foi utilizado para avaliar a competência para os cuidados iniciais (pré-teste). Este processo de avaliação foi realizado uma vez com cada paciente e teve duração de 30 a 40 minutos.

*Etapa 2. Preparação para a transição do hospital para o domicílio*: após a avaliação da preparação para a transição e das variáveis contextuais, iniciou-se a preparação através de processos educativos. A intervenção *Plan Cuidarte* inclui um componente educativo robusto, ministrado em duas sessões: a primeira aborda a dimensão do *Conhecimento* (uma sessão de 20 a 30 minutos), enquanto a segunda aborda as dimensões *Instrumental* e *Antecipação* (uma sessão de 30 a 40 minutos); sendo estas as dimensões da *competência para o cuidado*. No final de cada sessão educativa, foram utilizadas questões orientadoras para avaliar a compreensão do material, seguindo o método *Teach Back* (TB) para identificar e corrigir lacunas ou erros na informação. Este método mostrou-se capaz de melhorar múltiplos resultados em pacientes com IC^20^. Nesta fase, foram utilizados um folheto e um módulo de aprendizagem digital; as sessões educativas incluíram a definição de insuficiência cardíaca, monitorização de sinais e sintomas, adesão ao tratamento farmacológico e não farmacológico, identificação de sinais de alerta e tomada de decisão de acordo com as recomendações nacionais e internacionais para o cuidado da IC.

*Padrões de resposta*: este conceito é utilizado para determinar se o resultado de uma transição é saudável ou não após a implementação da intervenção. O *Plan Cuidarte* foi avaliado utilizando os seguintes indicadores de resposta: satisfação do paciente com a intervenção no que tange à preparação da transição do hospital para casa e competência para o cuidado após a intervenção (pós-teste).

A intervenção foi realizada presencialmente e de forma individual durante a hospitalização; em ambos os grupos os participantes continuaram recebendo, de forma paralela, os cuidados clínicos correspondentes. A intervenção foi conduzida por assistentes de pesquisa, estudantes de Enfermagem treinados neste processo.

O Grupo Comparação, por outro lado, recebeu o atendimento padrão na alta hospitalar, que consistiu no fornecimento das instruções gerais de cuidados no dia da alta. Os participantes receberam uma explicação sobre as recomendações de alta, incluindo o processo administrativo para solicitar consultas de acompanhamento, procedimentos e medicamentos. Além disso, uma enfermeira explicou as precauções necessárias para o uso dos medicamentos prescritos.

### Processamento e análise de dados

Foram realizadas análises por intenção de tratar e por protocolo. Os dados armazenados em planilhas do Excel foram analisados por meio do *software* SPSS, versão 19. As variáveis sociodemográficas foram analisadas via estatística descritiva, sendo o teste qui-quadrado utilizado para comparações. No que se refere à variável “competência para o cuidado”, a comparação intragrupo foi realizada com o teste de Wilcoxon e a comparação intergrupos com o teste U de Mann-Whitney. A análise do tamanho do efeito foi realizada com o *d* de Cohen e, dada a dimensão da amostra, foi efetuado um ajuste utilizando o *g* de Hedges. A satisfação do paciente com a intervenção recebida foi analisada através de percentagens.

### Considerações éticas

O estudo foi conduzido de acordo com as diretrizes éticas nacionais e internacionais e foi aprovado pelo Comitê de Ética da Universidad de Caldas em 24 de fevereiro de 2023, sob o número CBCS-004. Foi autorizado pelas instituições de saúde participantes. Cada participante foi informado sobre o objetivo e os detalhes do estudo e forneceu seu consentimento livre e esclarecido.

Com relação à identificação de potenciais danos, foi realizada uma avaliação não sistemática, dado o risco mínimo da pesquisa. Uma análise preliminar foi conduzida, considerando como risco potencial que algumas questões dos instrumentos pudessem gerar sentimentos de ansiedade, tristeza, medo ou incerteza, entre outros. Consequentemente, esse aspecto foi incluído no Termo de Consentimento Livre e Esclarecido, especificando ao participante a possibilidade de expressar espontaneamente tais sentimentos, receber o apoio necessário e desistir do estudo, caso assim desejasse.

## Resultados

O relatório do estudo seguiu as diretrizes do *Consolidated Standards of Reporting Trials* (CONSORT)^21^ para relatar ensaios clínicos randomizados. A elegibilidade de 150 participantes foi avaliada; 42 não atenderam aos critérios de inclusão e 8 recusaram-se a participar, resultando em uma taxa de recrutamento de 92%. Consequentemente, 100 participantes foram randomizados (50 para cada grupo) e analisados por intenção de tratar. Uma análise secundária, por protocolo, também foi realizada com uma amostra de 46 participantes do Grupo Experimental e 37 do Grupo Comparação (participantes que concluíram o estudo), com uma eficiência de amostragem de 98%. Observou-se uma taxa de perda de seguimento de 17% (Figura 1).

**Figura 1:** Fluxograma^21^ de participantes

### Características demográficas

A idade média dos pacientes era de 70±10,8 anos para o Grupo Experimental e 69,8±11,1 anos para o Grupo Comparação (*p=*0,199) (Teste U de Mann-Whitney). Por outro lado, 48% dos pacientes do Grupo Experimental e 60% do Grupo Comparação residiam na capital do departamento de Caldas, Colômbia. Os participantes tinham um mínimo de 1 mês e um máximo de 30 anos desde o diagnóstico de IC. Não foram encontradas diferenças estatisticamente significativas (*p*>0,05) na comparação das características demográficas entre os dois grupos, indicando a comparabilidade inicial dos grupos para essas variáveis (Tabela 1).

**Tabela 1:** Variáveis sociodemográficas correspondentes aos pacientes dos grupos Experimental e de Comparação (n = 100). Colômbia, 2024

Variáveis	Categorias	Grupo Experimental		Grupo Comparação		p*
		n=50		n=50		
		Frequência	Percentagem	Frequência	Percentagem	
Sexo	Masculino	30	60	32	64	0,680*
	Feminino	20	40	18	36	
Educação	Sem estudos	5	10	2	4	0,711*
	Ensino Fundamental Incompleto	20	40	19	38	
	Ensino Fundamental Completo	6	12	5	10	
	Ensino Médio Incompleto	9	18	11	22	
	Ensino Médio Completo	5	10	9	18	
	Técnico/Tecnólogo	1	2	2	4	
	Ensino Superior Completo	4	8	2	4	
Estado civil	Solteiro	12	24	10	20	0,560*
	Casado	18	36	25	50	
	Separado/Divorciado	4	8	2	4	
	Viúvo	10	20	10	20	
	União estável	6	12	3	6	
Ocupação	Do lar	29	58	30	60	0,787*
	Funcionário	3	6	5	10	
	Trabalhador independente	13	13	12	24	
	Outro	5	5	3	6	
Estrato socioeconômico	1 (Baixo)	18	36	12	24	0,140*
	2	17	34	16	32	
	3	6	12	17	34	
	4	4	8	2	4	
	5	1	2	0	0	
	6 (Alto)	4	8	3	6	
Possui um único cuidador.	Sim	36	72	43	86	0,140 ^†^
	Não	14	28	7	14	

*Valor *p* obtido com o teste do qui-quadrado; ^†^Valor *p* obtido com o teste exato de Fisher

### Percepção da carga e do apoio e utilização das TICs

Em relação à percepção da carga e do apoio ao cuidado, a satisfação com o apoio foi ligeiramente maior no Grupo Comparação em todas as dimensões avaliadas (apoio psicológico, familiar, religioso, econômico e social), embora não tenham sido encontradas diferenças estatisticamente significativas entre os grupos, o que confirmou sua comparabilidade inicial em relação a essas variáveis: apoio psicológico (*p=*0,201), apoio familiar (*p=*0,085), apoio religioso (*p=*0,194), apoio econômico (*p=*0,234) e apoio social (*p=*0,412).

Em relação ao uso das TICs para o cuidado, o Grupo Experimental apresentou baixos níveis de conhecimento, acesso e uso de computadores e internet, com mais de 62% em níveis baixos. No que tange aos telefones celulares, o desempenho foi melhor, com níveis conhecimento, acesso e uso, oscilando entre médio e alto, embora não totalmente adotados. A mídia tradicional, como televisão e rádio, apresentou altos níveis de conhecimento e acesso (cerca de 56%). Um padrão semelhante, embora ligeiramente mais favorável, foi observado no Grupo Comparação; no entanto, também predominaram baixos níveis de uso de computadores e internet (70% de conhecimento e 72% de acesso). Os telefones celulares apresentaram maior adoção, com quase 50% apresentando níveis altos de acesso e conhecimento, e nível de uso predominantemente médio. Em ambos os grupos, a adoção limitada de computadores e internet se repetiu, em contraste com a maior adoção de telefones celulares e os níveis elevados de utilização de televisão e rádio.

### Competência para o cuidado intragrupo

Na comparação entre o pré-teste e o pós-teste intragrupo, houve mudanças no Grupo Experimental, indicando uma melhora significativa (*p=*0,000) na competência para o cuidado, passando de um nível baixo para um nível médio; sendo que a competência de cuidado permaneceu em um nível baixo no Grupo Comparação (Tabela 2).

**Tabela 2:** Comparações intragrupo entre participantes dos grupos Experimental e de Comparação (n = 100). Colômbia, 2024

**Dimensões da competência para* *o* *cuidado**	Grupo Experimental (n=50)						
	Pré-teste*			Pós-teste^†^			*p* ^*‡*^
	Média	DP^§^	Qualificação	Média	DP^§^	Qualificação	
Conhecimento	1,84	2.034	Baixo	6.26	2.761	Médio	0,000
Singularidade	8.4	1.884	Médio	8,54	3.138	Médio	0,036
Instrumental	2.1	2.261	Baixo	5,56	2.442	Médio	0,000
Satisfação com grau de bem-estar	7,38	2.089	Médio	7.3	3.303	Médio	0,811
Antecipação	1.12	1,48	Baixo	3,56	1.875	Médio	0,000
Redes de apoio e relações	9.04	2.515	Médio	9,62	3.458	Médio	0,134
Competência geral para o cuidado	29,88	8.178	Baixo	40,84	14.148	Médio	0,000
**Dimensões da competência para* *o* *cuidado**	**Grupo Comparação (n=50)**						
	Pré-teste*			**Pós-teste^†^**			***p* ^*‡*^**
	**Média**	**DP^§^**	**Qualificação**	**Média**	**DP^§^**	**Qualificação**	
Conhecimento	1,94	1.921	Baixo	2.4	2.109	Baixo	0,154
Singularidade	8,74	2.174	Médio	6,5	4.464	Baixo	0,009
Instrumental	2.22	1.765	Baixo	2.6	2.312	Baixo	0,277
Satisfação com grau de bem-estar	6,9	1.876	Médio	5,74	4.309	Médio	0,074
Antecipação	1.22	1.282	Baixo	1,66	1.547	Baixo	0,122
Rede de apoio e relações	9,62	2.506	Médio	7,28	4.656	Médio	0,005
Competência geral para o cuidado	30,64	6.03	Baixo	26.18	16.999	Baixo	0,697

*Antes da intervenção; ^†^Após a intervenção; ^‡^Valor *p* obtido com o teste de Wilcoxon; ^§^Desvio-Padrão

### Competência para o cuidado intergrupo

Em relação à competência para o cuidado, houve diferenças estatisticamente significativas entre o Grupo Comparação e o Grupo Experimental ao final da intervenção. Os pacientes do Grupo Experimental demonstraram melhor competência para o cuidado nas dimensões de conhecimento, instrumental, antecipação (*p=*0,000), singularidade (*p=*0,036) e relações (*p=*0,005), bem como na competência geral para o cuidado (*p*=0,000); na dimensão de satisfação com o grau de bem-estar houve melhora no Grupo Experimental, embora ela não tenha sido estatisticamente significativa (Tabela 3).

**Tabela 3:** Comparação intergrupos dos participantes dos grupos Experimental e Comparação (n = 100). Colômbia, 2024

Dimensões da competência para o cuidado	Grupo Experimental (n=50)		Grupo Comparação (n=50)		*p* ^*†*^
	Média	DP*	Média	DP*	
Conhecimento	6.26	2.761	2.4	2.109	0,000
Singularidade	8,54	3.138	6,5	4.464	0,036
Instrumental	5,56	2.442	2.6	2.312	0,000
Satisfação com o grau de bem-estar	7.3	3.303	5,74	4.309	0,076
Antecipação	3,56	1.875	1,66	1.547	0,000
Rede de apoio e relações	9,62	3.458	7,28	4.656	0,005
Competência geral para o cuidado	40,84	14.148	26.18	16.999	0,000

*Desvio-Padrão; ^†^Valor *p* obtido com o teste U de Mann-Whitney

Observou-se um grande tamanho de efeito em favor do Grupo Experimental (d de Cohen=0,94; g de Hedges=0,93; IC 95%: 0,52-1,35), indicando uma diferença clinicamente relevante entre os dois grupos no desfecho analisado. Na comparação entre o Grupo Experimental (Média=40,84, Desvio-Padrão=14,14, tamanho da amostra=50) e o Grupo Comparação (Média=26,18, Desvio-Padrão=16,99, tamanho da amostra=50). Este resultado indica uma diferença substancial entre os grupos, com grande magnitude de efeito que sugere relevância prática na variável analisada^22^ ^-^ ^23^.

Adicionalmente, foi realizada uma análise de dados por protocolo, incluindo os participantes que concluíram a intervenção. Um tamanho de efeito grande (d de Cohen*=*1,19 e Intervalo de Confiança de 95%) foi observado ao comparar o Grupo Experimental (Média=44,39, Desvio-Padrão=7,54, tamanho da amostra=46) e o Grupo Comparação (Média=35,38, Desvio-Padrão=7,67, tamanho da amostra=37). Esse resultado indica que a intervenção teve um efeito positivo.

Considerando o tamanho da amostra do presente estudo, foi realizado um ajuste através do cálculo do *g* de Hedges, visto que este fornece uma estimativa não enviesada do tamanho do efeito, especialmente adequada para amostras de tamanho finito. Obteve-se um valor de *g=*1,18 [Intervalo de Confiança de 95% (0,71, 1,64)], indicando um efeito de grande magnitude da intervenção na competência para o cuidado.

### Satisfação com a intervenção para preparar a transição do hospital para o domicílio

A maioria dos participantes do Grupo Experimental classificou a intervenção do *Plan Cuidarte* como excelente, destacando a qualidade da educação oferecida sobre o cuidado da insuficiência cardíaca, as ferramentas utilizadas e a atitude da equipe que a ministrou (74%, 80% e 76% dos participantes classificaram essas características como excelentes, respectivamente). Já no Grupo Comparação, a maioria dos participantes classificou o processo de preparação para a alta hospitalar como bom, apontando a necessidade de aprimorar aspectos como o uso de ferramentas educativas.

## Discussão

Este estudo avaliou o efeito da intervenção de Enfermagem *Plan Cuidarte*, implementada durante a transição do hospital para o domicílio, na competência de cuidado de indivíduos com IC, comparando-a ao cuidado convencional na alta hospitalar. Os indivíduos que receberam a intervenção *Plan Cuidarte* apresentaram melhorias estatisticamente significativas em relação ao Grupo Comparação nas dimensões de competência para o cuidado relacionadas ao conhecimento, à implementação e à antecipação, bem como em seu nível de competência geral para o cuidado.

A literatura indica que as intervenções de Enfermagem baseadas em teoria produzem melhores resultados do que as amparadas em cuidados convencionais^24^. Para este estudo, a Teoria das Transições permitiu a estruturação da intervenção *Plan Cuidarte*, que superou o plano de alta convencional. Da mesma forma, a estrutura de Whittemore^18^ facilitou o planejamento e a avaliação desta intervenção estruturada, que apresenta potencial para ser replicada a fim de promover a prática baseada em evidências.

Com base nessas descobertas e graças à educação do paciente como suporte para melhorar e controlar sua doença, espera-se que as pessoas tratadas com o *Plan Cuidarte* tenham uma transição tranquila do hospital para o domicílio, uma vez que, conforme documentado, o acima mencionado é crucial para melhorar os desfechos em pacientes com IC^25^.

Esses resultados confirmam os de outros estudos nacionais realizados em pacientes com doenças crônicas que relataram que os planos de alta estruturados melhoram essa competência^8^.

Esta mudança positiva alcançada no nível de competência para o cuidado do *Plan Cuidarte* responde à necessidade de fortalecer este grupo de pacientes, considerando que, antes da implementação de um programa de alta para pessoas com doença cardiovascular, a competência deles para o cuidado não era a ideal para gerenciar sua condição de saúde^8^.

Esta pesquisa também confirma achados sobre o perfil epidemiológico de pacientes com insuficiência cardíaca relatados em estudos anteriores. Neste caso, bem como em outros documentados, a maioria dos pacientes diagnosticados com IC são idosos e do sexo masculino^6^ ^,^ ^26^ ^-^ ^28^.

Os resultados relatados na presente pesquisa sobre escolaridade, estado civil, estrato socioeconômico e presença de um único cuidador também coincidem com estudos anteriores^5^.

Além disso, os desfechos revelam uma lacuna significativa de alfabetização digital, especialmente em relação às tecnologias que exigem habilidades técnicas avançadas; isso reforça a necessidade de fortalecer o treinamento no uso de computadores e da internet. Um estudo sobre a experiência de pacientes com IC que utilizam as TICs para o autogerenciamento relata que essa baixa alfabetização pode levar a experiências negativas e, portanto, recomenda o fornecimento de suporte profissional ao incorporar novas tecnologias em seus cuidados^29^. Esse suporte deve ser considerado um elemento significativo a ser incluído na assistência à saúde desses indivíduos, uma vez que o uso das TIC na transição de cuidados de pacientes com insuficiência cardíaca pode melhorar sua qualidade de vida e satisfação com o atendimento, além de reduzir as reinternações hospitalares^30^.

É importante notar que 3% dos pacientes hospitalizados faleceram durante o curso deste estudo. Isso está de acordo com os achados do estudo *Recolfaca*, que relata que 3% a 10% dos pacientes hospitalizados por IC morrem no hospital^5^. Essa alta taxa de mortalidade reforça ainda mais a necessidade de desenvolver intervenções para prevenir e controlar descompensações, bem como para reduzir o número de reinternações hospitalares em pacientes com IC^6^.

No que diz respeito às implicações desta pesquisa para a prática e estudos futuros, é essencial considerar que a insuficiência cardíaca é a principal causa de hospitalização em pessoas com mais de 65 anos de idade^31^. Portanto, é pertinente avaliar o efeito do *Plan Cuidarte* em desfechos clínicos como reinternações hospitalares, visitas ao pronto-socorro e mortalidade. Consequentemente, uma abordagem longitudinal é necessária para analisar o impacto das intervenções ao longo do tempo e em diferentes populações, especialmente em idosos, que são mais vulneráveis a complicações após a alta hospitalar^32^. Conforme proposto, esse acompanhamento poderia incorporar ferramentas de telessaúde, telemonitoramento, acompanhamento telefônico ou visitas domiciliares^6^, o que implicaria a integração do treinamento digital como parte do processo de cuidado.

Da mesma forma, em estudos subsequentes será importante analisar a eficácia do *Plan Cuidarte* incluindo cuidadores de pacientes com IC, visto que intervenções destinadas à alta hospitalar demonstraram melhorar a competência para o cuidado da díade paciente/cuidador^8^.

Os resultados deste estudo destacam a importância de continuar a desenvolver e implementar programas de intervenção de Enfermagem estruturados e personalizados que melhorem a competência nos cuidados a pacientes com IC. Estes programas devem ser integrados ao processo de alta hospitalar assegurando uma transição fluida para os cuidados ambulatoriais de forma frequente e contínua, sob a liderança de profissionais de Enfermagem^33^.

Além disso, aprimorar as habilidades de autocuidado do paciente é fundamental para o manejo da doença, visando os melhores resultados clínicos possíveis e uma experiência positiva no cuidado^34^. O *Plano Cuidarte* pode ser uma ferramenta útil, rigorosa e abrangente para pacientes com IC que devem continuar o tratamento em casa.

Apesar dos resultados obtidos, é importante ressaltar que a autopercepção da competência foi coletada por meio de autorrelato e avaliada em um curto período de acompanhamento. Tal fator limita a determinação de implicações práticas e da relação custo-benefício em desfechos clínicos e pós-alta, tais como qualidade de vida, estado geral de saúde, complicações, reinternações, tempo de internação e taxas de mortalidade, entre outros. Da mesma forma, é importante notar que esses resultados se referem a um grupo específico de pacientes e que o estudo deve ser replicado para que possam ser generalizados para outras populações ou contextos.

## Conclusão

O *Plan Cuidarte* é uma intervenção sistemática e teoricamente sólida que, quando aplicada durante a hospitalização, melhora significativamente a competência para o cuidado de pessoas com insuficiência cardíaca durante o período de transição do hospital para o domicílio.

Sua estrutura metodológica e fundamentação nos próprios referenciais conceituais da disciplina sustentam sua replicabilidade e relevância para integração na prática clínica de rotina. Os resultados demonstram que a participação ativa dos profissionais de Enfermagem é essencial para promover uma transição mais segura e contribuir para um melhor manejo da doença e desfechos de saúde mais favoráveis. Assim, o estudo reafirma o valor estratégico das intervenções de enfermagem durante a alta hospitalar e fornece evidências que fortalecem práticas eficazes e sustentáveis no cuidado de pessoas com doenças crônicas, como a insuficiência cardíaca.

## Data Availability

O conjunto de dados deste artigo está disponível no link https://doi.org/10.6084/m9.figshare.29361920.v1

# Translation: Efecto de un plan de transición del hospital al hogar para personas con Insuficiencia Cardíaca: ensayo clínico aleatorio^*^

**DOI:** 10.1590/1518-8345.8271.4941

**Authors:** Claudia Marcela Vallejo-Ramírez, John Edison Cárdenas-Galvis, Gloria Carvajal-Carrascal, Alejandra Fuentes-Ramírez, Beatriz Sánchez-Herrera

**Affiliations:** 1 Universidad de Caldas, Facultad de Ciencias para la Salud, Manizales, Caldas, Colombia.; 2 Universidad de La Sabana, Facultad de Enfermería y Rehabilitación, Chía, Cundinamarca, Colombia.

**Keywords:** Enfermería, Transición del Hospital al Hogar, Alta del Paciente, Insuficiencia Cardiaca, Educación en Salud, Teoría de Enfermería.

## Abstract

**Objetivo:**

determinar el efecto del Plan Cuidarte sobre la competencia para el cuidado de las personas con insuficiencia cardiaca durante el periodo de transición del hospital al hogar.

**Método:**

ensayo clínico aleatorizado del tipo pretest-postest que incluyó 100 participantes (Grupo Experimental=50, Grupo Comparación=50). El Grupo Experimental recibió el Plan Cuidarte, que contiene dos etapas: la primera consiste en evaluar la preparación para el egreso y la segunda en la preparación para el egreso a través de procesos de educación. El Grupo Comparación recibió el plan convencional de egreso. Se realizó un análisis por intención de tratar con comparaciones intra e intergrupo y la variable dependiente fue la *competencia para el cuidado* medida con el instrumento Cuidar. Los datos fueron analizados con estadística descriptiva y analítica, siguiendo los principios éticos en investigación.

**Resultados:**

los participantes del Grupo Experimental presentaron una mejoría estadísticamente significativa en la competencia para el cuidado respecto al Grupo Comparación, con un tamaño de efecto grande.

**Conclusión:**

el Plan Cuidarte mejora la competencia para el cuidado de las personas con insuficiencia cardiaca durante la transición del hospital al hogar. Basada en la teoría de las transiciones, esta intervención tiene el potencial de ser implementada en la preparación para el egreso en esta población. Protocolo del ensayo registrado en febrero del 2024, título del protocolo: *Effect of Plan Cuidarte on the Care Competence of People with Heart Failure*, número de identificación NCT06309875 disponible en: https://clinicaltrials.gov/study/NCT06309875.

## Destacados

**(1)** El Plan Cuidarte mejora la competencia para el cuidado en Insuficiencia Cardíaca.

**(2)** El Plan Cuidarte mostró un efecto grande en la competencia para el cuidado.

**(3)** La intervención fue efectiva en la transición del hospital al hogar en IC.

**(4)** El rol del enfermero es fundamental en el egreso hospitalario anticipado.

**(5)** El Plan Cuidarte se basa en teoría de Enfermería aplicada.

## Introducción

Las enfermedades cardiovasculares (ECV) son la causa más frecuente de mortalidad del mundo, con mayor incidencia en países de bajos y medianos ingresos^1^. Una de las expresiones de ECV es la insuficiencia cardíaca (IC): aproximadamente 1 de cada 4 personas desarrollará IC a lo largo de su vida^2^. La IC en Latinoamérica presenta una alta carga de enfermedad, con prevalencia y mortalidad elevadas, considerándose un problema de salud pública y con una prevalencia del 1% en esta región^3^. También es importante considerar que los pacientes con IC presentan altas tasas de reingresos hospitalarios: en un estudio realizado en Brasil, las personas con IC presentaron reingresos hospitalarios del 26,2% a los 90 días, con una tasa de mortalidad intrahospitalaria del 10,9% e incidencia acumulada de mortalidad a los 12 meses del 27,7%^4^.

En Colombia, la prevalencia de la IC es del 2,3%, afectando aproximadamente a 1.097.201 personas^5^. El costo de la hospitalización a nivel nacional casi duplica el de la atención ambulatoria^6^. Los pacientes con IC se ven frecuentemente expuestos a ingresos y reingresos hospitalarios; en consecuencia, se enfrentan continuamente al proceso de transición del hospital al hogar y se ha documentado que, en este proceso, presentan retos como dificultades con el uso de la medicación, hábitos nocivos como fumar y consumir alcohol, exposición a situaciones estresantes y, finalmente, se ha reportado un seguimiento profesional insuficiente durante la transición del hospital al hogar^7^.

En tal sentido, es necesario implementar estrategias que mejoren la atención de las personas con IC la cual, por su condición de enfermedad crónica, exige que quienes la padecen sean competentes para cuidarse. Estudios realizados en Colombia han reportado que las personas con enfermedades crónicas no tienen una competencia óptima para el cuidado^8^. Adecuados niveles de competencia para el cuidado son fundamentales a fin de mantener la seguridad durante este proceso de transición^7^, dado que se trata de un momento vulnerable para las personas con IC caracterizado por altas tasas de reingresos y de mortalidad hospitalaria. Este periodo exige una preparación integral centrada en el paciente y brindada antes del egreso^9^.

Dada la actual tendencia a minimizar la duración de la estadía hospitalaria, los riesgos son mayores principalmente durante el periodo de los tres primeros meses después de una hospitalización por IC^6^; así como su costo personal, social y económico, su cronicidad hace necesario implementar intervenciones específicas para este grupo poblacional y que mejoren su competencia para el cuidado.

La transición del hospital al hogar es un fenómeno de Enfermería que se origina en la Teoría de las Transiciones de Meleis; es un proceso sistémico, vulnerable y multidimensional y es necesario diseñar intervenciones complejas para abordarla. La transición del hospital al hogar se ha denominado “transiciones de atención”: períodos críticos, especialmente en la población con IC, que requiere una cuidadosa gestión para prevenir reingresos y mejorar su evolución^10^.

Esta investigación integra tres conceptos de la teoría de las transiciones: Condiciones para la transición, Terapéutica de Enfermería y Patrones de respuesta^11^. Las condiciones para la transición influyen en el logro de una transición saludable (meta del cuidado según esta teoría). Es fundamental considerarlas en el diseño de intervenciones personalizadas, ya que se ha demostrado que una preparación para el egreso individualizada y ajustada a las necesidades específicas mejora los resultados en pacientes con IC^12^. El desarrollo de las intervenciones de Enfermería dirigidas a la preparación para la transición se enmarca en el concepto de Terapéutica de Enfermería.

Se ha recomendado que, como parte del tratamiento no farmacológico, se incluyan intervenciones de educación al paciente en aspectos como dieta, actividad física y manejo del estrés, entre otros^13^.

Es por esto que la Enfermería ha buscado diversos mecanismos para apoyar el cuidado de las personas con IC, mejorar su situación de salud y evitar las complicaciones con una adecuada planificación para la transición del hospital al hogar. Una revisión de las intervenciones de Enfermería para cuidar a personas con IC durante esta transición descubrió que la educación y el seguimiento de Enfermería favorecen la independencia en el cuidado y disminuyen los reingresos y la mortalidad. Sin embargo, reportan las autoras que estas intervenciones no se utilizan con frecuencia, que la educación no tiene el nivel de especialización necesario y que no se encuentran estandarizadas^14^. Otro estudio, que revisó programas multicomponentes con intervenciones de educación y seguimiento, detectó que estos redujeron la mortalidad, los reingresos y los días de estancia^6^. Se suma a estos el reporte de planes de egreso dirigidos a pacientes con IC buscando fortalecer su competencia para el cuidado^15^; sin embargo, y a pesar de estos avances, la evidencia aún es débil.

Además del diseño de intervenciones de Enfermería que atiendan la transición del hospital al hogar, es fundamental medir y socializar su impacto; por lo anterior, un subconcepto de la teoría utilizado es el de indicadores de resultados, el cual permite determinar si el resultado de una transición es saludable o no, donde los individuos demuestran dominio de las habilidades y los comportamientos necesarios para manejar sus nuevas situaciones. Es a partir de estos indicadores que se encuentran los resultados de salud centrados en el paciente como lo son la satisfacción y las habilidades de cuidado^10^.

Dada la alta incidencia y el costo personal, social y económico de las personas con IC que son atendidas en tres instituciones del departamento de Caldas, Colombia, fue necesario estructurar un plan para la atención de la transición del hospital al hogar específico para ellos.

Por lo anterior, para el presente estudio se planteó la siguiente hipótesis de investigación: la intervención Plan Cuidarte (basada en el concepto teórico de la Terapéutica de Enfermería) modifica positivamente la competencia para el cuidado, con un aumento de al menos 5 puntos (Subconcepto: Indicador de resultado), de las personas con IC que se encuentran en la transición del hospital al hogar en tres hospitales de Manizales, comparado con la atención de egreso convencional, donde el Plan Cuidarte es el acrónimo de Plan de Cuidado Anticipado y Estructurado para la atención de la transición del hospital al hogar de pacientes con IC.

El presente estudio tuvo como objetivo determinar el efecto del Plan Cuidarte sobre la competencia para el cuidado de las personas con IC durante el periodo de transición del hospital al hogar.

## Método

### Diseño del estudio

Ensayo controlado aleatorizado (ECA) paralelo, de superioridad, con Grupo Comparación y medición pre y post intervención; contó con prueba piloto previa que permitió ajustar el protocolo inicial. Imposibilidad de cegamiento de los participantes debido a la característica educativa de la intervención; sin embargo, se mantuvo el cegamiento de los analistas de la información proporcionándoles datos desidentificados y codificados.

En la fase de preparación y diseño de la intervención, tanto los pacientes como el público en general apoyaron el diseño con la revisión y validación del material educativo y su forma de entrega pedagógica, lo que contribuyó a su mejor comprensión y aceptación. Las instituciones revisaron el protocolo y apoyaron su cualificación.

### Lugar y período de recolección de datos

Período de reclutamiento y seguimiento de marzo a agosto 2024, en tres instituciones de salud de la ciudad de Manizales, Caldas, Colombia.

### Población

Personas mayores de 18 años hospitalizadas con diagnóstico de IC.

### Criterios de selección

Se incluyeron personas mayores de 18 años que ingresaron al servicio de hospitalización con diagnóstico de IC en 3 centros hospitalarios, sin alteración mental o funcional que impidiera autonomía en su cuidado. El diagnóstico de IC se determinó mediante revisión de la historia clínica y confirmación con el personal de salud a cargo del paciente. Tanto la condición cognitiva como el nivel de funcionalidad se evaluaron a partir de dos componentes de la Encuesta de caracterización para el cuidado de la diada cuidador-persona con enfermedad crónica (GCPC-UN-D)^16^, que fueron el cuestionario *Short Portable Mental Status Questionnaire* (SPMSQ - PFEFFER) y la escala PULSES, acrónimo correspondiente a *stability of the Pathology* (estabilidad de la patología), *Use of upper limbs* (utilización de miembros superiores), *Locomotion* (locomoción), *Sensory capacity* (capacidad sensorial), *Elimination* (eliminación) y *Social activity* (actividad social). Se excluyó a personas candidatas a trasplante cardiaco en los próximos 6 meses y a quienes fueran trasladados a cuidados intensivos o intermedios. Los centros hospitalarios incluidos debían atender de manera integral a personas con IC.

### Definición de la muestra

El muestreo fue no probabilístico, por conveniencia. El tamaño de muestra se calculó considerando errores tipo I y tipo II en 0,05, tamaño del efecto (d de Cohen) en 0,8 y potencia de 95%, conociendo la atrición estimada en un 20% de acuerdo con lo reportado en el estudio piloto previo. El cálculo muestral inicial sugirió 100 participantes en la muestra, distribuidos entre los grupos Experimental y Comparación con 50 sujetos cada uno. Se realizó asignación aleatoria 1:1 tanto al Grupo Experimental como al Comparación, la unidad de aleatorización fueron los participantes del estudio, el proceso se realizó a través de un tercero, apoyado de un generador informático utilizando aleatorización por permutación. Los asistentes de investigación, quienes asignaron los participantes a los grupos, desconocían la secuencia dado que solo podían conocer el grupo de asignación a través de su teléfono móvil al momento de la asignación.

### Variables del estudio

Se evaluó como resultado primario el nivel de competencia para el cuidado, variable dependiente del estudio, definida de la siguiente manera: la capacidad, habilidad y preparación que tiene el paciente para ejercer el cuidado. Comprende seis dimensiones: Conocimiento (Nociones respecto a la enfermedad), Unicidad (Herramientas para hacer frente a los desafíos), Instrumental (Habilidades para realizar las labores de cuidado), Disfrute (Grado de bienestar y satisfacción personal), Anticipación (Nociones para anticiparse a las necesidades futuras), y Relación social e interacción (Relaciones sociales y redes de apoyo)^17^. Como *resultado secundario* se evaluó la satisfacción con la intervención. Como variables independientes se midieron variables contextuales como conocimiento, acceso y uso de las Tecnologías de la Información y Comunicación (TIC) para el cuidado, percepción de carga y apoyo en el cuidado, además de realizarse la caracterización demográfica.

### Instrumentos utilizados y recolección de datos

La caracterización inicial realizada en la primera fase del *Plan Cuidarte* se llevó a cabo con la encuesta GCPC-UN-D, validada en Colombia^16^; de esta, se aplicaron las preguntas orientadas al paciente con las cuales se determinó la caracterización demográfica, la percepción de carga y apoyo, y el empleo de las TIC.

La competencia para el cuidado se midió a través del instrumento Cuidar (versión paciente corta) validada en Colombia, cuyo alfa de Cronbach fue de 0,928^17^. Cuidar incluye las seis dimensiones de la competencia para el cuidado, que son valoradas mediante 20 preguntas calificadas con escala Likert. La competencia para el cuidado se puede categorizar en alta (49-60), media (37-48) o baja (0-36) y se midió previo al inicio de la intervención o atención habitual (T0) y luego de la intervención o atención habitual (T1); para el análisis y las comparaciones, se utilizó la media de las dimensiones y de la competencia general para el cuidado.

Por último, se valoró la satisfacción con la intervención para el egreso hospitalario, medida luego de la intervención en el Grupo Experimental o después de la atención habitual en el Grupo Comparación (T1) con una encuesta desarrollada *ad hoc* para el presente estudio, por lo cual aún no cuenta con estudios de validación.

### Intervención y comparación

El Grupo Experimental recibió la intervención Plan Cuidarte para personas con IC. Se trata de una intervención de Enfermería dirigida a atender la transición del hospital al hogar de esta población. El Plan Cuidarte atendió los parámetros para el desarrollo de intervenciones sistemáticas de Whittemore^18^. Para garantizar la fidelidad en el desarrollo del protocolo se diseñó un manual de intervención en el que se especificó la secuencia para implementar la intervención, la aplicación e interpretación de los instrumentos y una lista de verificación para evaluar la fidelidad, logrando que la intervención se administrara como estaba prevista.

El diseño de la intervención estuvo orientado conceptualmente por la teoría de las transiciones^19^, como se describe a continuación.

### Aplicación de la teoría para el diseño de la intervención

Este estudio retomó la teoría de mediano rango de las transiciones de Afaf Ibrahim Meleis como marco para diseñar la intervención propuesta: Plan Cuidarte, como se describe a continuación.

*Naturaleza de la transición*: la transición del hospital al hogar se clasifica teóricamente como una transición de salud y enfermedad. Para las personas con IC, se ha descrito bajo el término de transición de atención^10^.

*Terapéutica de Enfermería*: son medidas que pueden aplicarse a las intervenciones terapéuticas durante las transiciones; especialmente, las intervenciones se sustentan en procesos de evaluación y educación^10^. Considerando la vulnerabilidad que implican las transiciones de atención en los pacientes con IC, se diseña la intervención *Plan Cuidarte* orientada a fortalecer la competencia para el cuidado del paciente durante la transición del hospital al hogar.

La intervención Plan Cuidarte se encuentra constituida por dos etapas orientadas por dos subconceptos teóricos:

*Etapa 1. Evaluación de la preparación para la transición del hospital al hogar*: pretende generar pleno conocimiento del paciente con IC respecto a su preparación para la transición a través del conocimiento de las *Condiciones para la transición* (subconcepto teórico). Para la evaluación de estas condiciones se aplicaron las preguntas correspondientes a la evaluación del paciente de la Encuesta GCPC-UN-D, que evalúa aspectos relacionados con las variables contextuales que pueden influir en el proceso de transición del hospital al hogar; en esta etapa también se utilizó el instrumento Cuidar (versión paciente) para la evaluación de la competencia para el cuidado inicial (pretest). Este proceso de evaluación se realizó una vez por cada paciente en 30-40 minutos.

*Etapa 2. Preparación para la transición del hospital al hogar*: una vez evaluada la preparación para la transición y las variables contextuales, se inició la preparación a través de procesos educativos. La intervención Plan Cuidarte cuenta con un sólido componente educativo realizado en 2 sesiones: la primera abordó la dimensión de *Conocimiento* (1 sesión de 20-30 minutos), mientras que la segunda abordó las dimensiones de *Instrumental* y *Anticipación* (1 sesión de 30-40 minutos); las anteriores son dimensiones de la *competencia para el cuidado*. Al final de cada sesión educativa se aplicaron las preguntas orientadoras para evaluar la comprensión de la educación, siguiendo los lineamientos del M*étodo Teach Back* (TB) para identificar y subsanar vacíos o errores en la información. Este método ha demostrado mejorar múltiples resultados en pacientes con IC^20^. En esta fase se empleó una cartilla y un módulo didáctico digital; las sesiones educativas incluyeron definición de la IC, seguimiento de los signos y síntomas, adhesión al tratamiento farmacológico y no farmacológico, identificación de los signos de alarma y toma de decisiones de acuerdo con las recomendaciones nacionales e internacionales para el cuidado de la IC.

*Patrones de respuesta*: este concepto se utiliza para determinar si el resultado de una transición es saludable o no después de implementar la intervención. El Plan Cuidarte fue evaluado por los siguientes indicadores de respuesta: satisfacción del paciente con la intervención de preparación para la transición del hospital al hogar y competencia para el cuidado luego de la intervención (postest).

La intervención se desarrolló de manera presencial e individualmente durante la hospitalización; los participantes de ambos grupos continuaron recibiendo de manera concomitante la atención clínica correspondiente. La intervención fue ejecutada por asistentes de investigación que eran estudiantes de Enfermería entrenados en este proceso.

Por su parte, el Grupo Comparación recibió la atención habitual de egreso, que consistió en proporcionar las indicaciones generales de cuidado el mismo día del egreso. Los participantes recibieron la explicación de las recomendaciones de egreso, incluyendo el proceso administrativo para la solicitud de citas de control, procedimientos y medicamentos. Además, una enfermera a cargo explicó los cuidados necesarios para el consumo de los medicamentos indicados.

### Procesamiento y análisis de los datos

Se realizó análisis por intención de tratar y por protocolo. Los datos se almacenaron en hojas de Excel y analizados en el programa SPSS, versión 19. Los datos sociodemográficos se analizaron con estadísticos descriptivos y se empleó la prueba de Chi-cuadrado para las comparaciones. En la variable “competencia para el cuidado”, la comparación intragrupo se desarrolló con la prueba de Wilcoxon y la intergrupo con la prueba U de Mann-Whitney. El análisis del tamaño del efecto se realizó con la *d* de Cohen y, dado el tamaño de muestra, se realizó un ajuste utilizando la *g* de Hedges. La satisfacción del paciente con la intervención recibida se manejó con análisis porcentual.

### Consideraciones éticas

El estudio se realizó de acuerdo con lineamientos de ética nacionales e internacionales y fue aprobado por el comité de ética de la Universidad de Caldas el 24 de febrero del año 2023 bajo consecutivo CBCS-004. Contó con la autorización de las instituciones de salud participantes. Se informó a cada uno de los participantes el propósito y los detalles del estudio y se obtuvo su consentimiento informado.

Respecto a la identificación de daños, se realizó una evaluación no sistemática, considerando que la investigación es de riesgo mínimo. Se ejecutó un análisis preliminar, considerando como riesgo potencial que algunas preguntas de los instrumentos pudieran generar sentimientos de ansiedad, tristeza, miedo o incertidumbre, entre otros. En consecuencia, se incluyó este aspecto en el consentimiento informado especificando al paciente la posibilidad de expresar de manera espontánea dichas sensaciones, de recibir la atención necesaria y de retirarse del estudio si así lo deseara.

## Resultados

El informe del estudio siguió los lineamientos *Consolidated Standards of Reporting Trials* (CONSORT)^21^ para informar ensayos clínicos aleatorizados. Se evaluó la elegibilidad de 150 participantes, de los cuales 42 no cumplieron los criterios de inclusión y 8 rechazaron participar, obteniendo una tasa de reclutamiento del 92%. En consecuencia, se aleatorizaron 100 participantes (50 a cada grupo) y se los analizó por intención de tratar. Asimismo, se realizó un análisis secundario por protocolo con una muestra compuesta por 46 personas del Grupo Experimental y 37 del Grupo Comparación (participantes que finalizaron el estudio), con una eficiencia muestral del 98%. Durante el seguimiento se obtuvo una tasa de pérdidas del 17% (Figura 1).

**Figura 1:** Diagrama de flujo^21^ de los participantes

### Características demográficas

La edad promedio de los pacientes fue 70**±**10,8 años para el Grupo Experimental y 69,8**±**11,1 años para el Grupo Comparación (*p*=0,199) (U de Mann-Whitney). El 48% de los pacientes del Grupo Experimental y el 60% del Grupo Comparación residía en la capital del departamento de Caldas, Colombia. Las personas participantes tenían un tiempo de diagnóstico con IC mínimo de 1 mes y máximo de 30 años. No se encontraron diferencias estadísticamente significativas (*p*>0,05) en la comparación de las características demográficas entre ambos grupos, lo que indicó la comparabilidad inicial de los grupos para estas variables (Tabla 1).

**Tabla 1:** Variables sociodemográficas correspondientes a los pacientes de los grupos Experimental y Comparación (n = 100). Colombia, 2024

Variables	Categorías	Grupo Experimental		Grupo Comparación		p*
		n=50		n=50		
		Frecuencia	Porcentaje	Frecuencia	Porcentaje	
Sexo	Masculino	30	60	32	64	0,680*
	Femenino	20	40	18	36	
Escolaridad	Sin estudios	5	10	2	4	0,711*
	Primaria Incompleta	20	40	19	38	
	Primaria Completa	6	12	5	10	
	Bachillerato Incompleto	9	18	11	22	
	Bachillerato Completo	5	10	9	18	
	Técnico/Tecnólogo	1	2	2	4	
	Universitaria Completa	4	8	2	4	
Estado civil	Soltero	12	24	10	20	0,560*
	Casado	18	36	25	50	
	Separado/Divorciado	4	8	2	4	
	Viudo	10	20	10	20	
	Unión Libre	6	12	3	6	
Ocupación	Hogar	29	58	30	60	0,787*
	Empleado	3	6	5	10	
	Trabajador independiente	13	13	12	24	
	Otro	5	5	3	6	
Estrato socioeconómico	1 (Bajo)	18	36	12	24	0,140*
	2	17	34	16	32	
	3	6	12	17	34	
	4	4	8	2	4	
	5	1	2	0	0	
	6 (Alto)	4	8	3	6	
Tiene un único cuidador	Si	36	72	43	86	0,140^†^
	No	14	28	7	14	

*Valor *p* obtenido con la prueba de Chi-cuadrado; ^†^Valor *p* obtenido con la prueba exacta de Fisher

### Percepción de carga y apoyo, y empleo de las TIC

Respecto a la percepción de carga y apoyo para el cuidado, la satisfacción con el apoyo fue ligeramente mayor en el Grupo Comparación en todas las dimensiones evaluadas (apoyo psicológico, familiar, religioso, económico y social), aunque no se encontraron diferencias estadísticamente significativas entre los grupos, lo que ratificó su comparabilidad inicial respecto a estas variables: apoyo psicológico (*p*=0,201), familiar (*p*=0,085), religioso (*p*=0,194), económico (*p*=0,234) y social (*p*=0,412).

En cuanto al empleo de las TIC para el cuidado, el Grupo Experimental mostró bajo conocimiento, acceso y uso de computadores e Internet, con más del 62% en niveles bajos. El celular tuvo mejores resultados, con niveles de conocimiento, acceso y uso entre medio y alto, aunque sin apropiación total. Los medios tradicionales como televisión y radio presentaron altos niveles de conocimiento y acceso (cerca del 56%). En el Grupo Comparación se observó un patrón similar, aunque ligeramente más favorable; no obstante, también predominaron los niveles bajos en computadores e Internet (70% en conocimiento y 72% en acceso). El celular mostró mayor apropiación, con cerca del 50% en niveles altos de acceso y conocimiento y un uso mayormente medio. En ambos grupos se repitió la limitada apropiación de computadores e Internet, frente a una mayor apropiación del celular y altos niveles de uso de televisión y radio.

### Competencia para el cuidado intragrupo

En la comparación entre los pretest y postest intragrupo hubo cambios en el Grupo Experimental, indicando una mejoría significativa (*p*=0,000) en la competencia para el cuidado, pasando de un nivel bajo a medio; la competencia para el cuidado se mantuvo en el nivel bajo en el Grupo Comparación (Tabla 2).

**Tabla 2:** Comparaciones intragrupo entre los participantes de los grupos Experimental y Comparación (n = 100). Colombia, 2024

Dimensiones de la competencia para el cuidado	Grupo Experimental (n=50)						
	Pretest*			Postest^†^			p^‡^
	Media	DE^§^	Calificación	Media	DE^§^	Calificación	
Conocimiento	1,84	2,034	Bajo	6,26	2,761	Medio	0,000
Unicidad	8,4	1,884	Medio	8,54	3,138	Medio	0,036
Instrumental	2,1	2,261	Bajo	5,56	2,442	Medio	0,000
Disfrute de condiciones de bienestar	7,38	2,089	Medio	7,3	3,303	Medio	0,811
Anticipación	1,12	1,48	Bajo	3,56	1,875	Medio	0,000
Red de apoyo y relaciones	9,04	2,515	Medio	9,62	3,458	Medio	0,134
Competencia global para el cuidado	29,88	8,178	Bajo	40,84	14,148	Medio	0,000
**Dimensiones de la competencia para el cuidado**	**Grupo Comparación (n=50)**						
	Pretest*			**Postest^†^**			**p^‡^**
	**Media**	**DE^§^**	**Calificación**	**Media**	**DE^§^**	**Calificación**	
Conocimiento	1,94	1,921	Bajo	2,4	2,109	Bajo	0,154
Unicidad	8,74	2,174	Medio	6,5	4,464	Bajo	0,009
Instrumental	2,22	1,765	Bajo	2,6	2,312	Bajo	0,277
Disfrute de condiciones de bienestar	6,9	1,876	Medio	5,74	4,309	Medio	0,074
Anticipación	1,22	1,282	Bajo	1,66	1,547	Bajo	0,122
Red de apoyo y relaciones	9,62	2,506	Medio	7,28	4,656	Medio	0,005
Competencia global para el cuidado	30,64	6,03	Bajo	26,18	16,999	Bajo	0,697

*Antes de la intervención; ^†^Después de la intervención; ^‡^Valor *p* obtenido con la prueba de Wilcoxon; ^§^Desviación Estándar

### Competencia para el cuidado intergrupo

En relación a la competencia para el cuidado, hubo diferencias estadísticamente significativas entre el Grupo Comparación y el Grupo Experimental al finalizar la intervención. Los pacientes del Grupo Experimental presentaron mejor competencia para el cuidado en las dimensiones de conocimiento, instrumental, anticipación (*p*=0,000), unicidad (*p*=0,036) y relaciones (*p*=0,005) y en la competencia global para el cuidado (*p*=0,000); en la dimensión “Disfrute de condiciones de bienestar” hubo una mejoría en el Grupo Experimental, aunque no fue estadísticamente significativa (Tabla 3).

**Tabla 3:** Comparaciones intergrupo de los participantes de los grupos Experimental y Comparación (n = 100). Colombia, 2024

Dimensiones de la competencia para el cuidado	Grupo Experimental (n=50)		Grupo Comparación (n=50)		p^†^
	Media	DE*	Media	DE*	
Conocimiento	6,26	2,761	2,4	2,109	0,000
Unicidad	8,54	3,138	6,5	4,464	0,036
Instrumental	5,56	2,442	2,6	2,312	0,000
Disfrute de condiciones de bienestar	7,3	3,303	5,74	4,309	0,076
Anticipación	3,56	1,875	1,66	1,547	0,000
Red de apoyo y relaciones	9,62	3,458	7,28	4,656	0,005
Competencia global para el cuidado	40,84	14,148	26,18	16,999	0,000

*Desviación Estándar; ^†^Valor *p* obtenido con la prueba U de Mann-Whitney

Se observó un tamaño del efecto grande a favor del Grupo Experimental (*d* de Cohen=0,94; *g* de Hedges=0,93; IC 95%: 0,52-1,35), lo que indica una diferencia clínicamente relevante entre ambos grupos en el desenlace analizado en la comparación entre el Grupo Experimental (Media=40,84, Desviación Estándar=14,14, tamaño de la muestra=50) y el Grupo Comparación (Media=26,18, Desviación Estándar=16,99, tamaño de la muestra=50). Este resultado indica una diferencia sustantiva entre los grupos, con magnitud de efecto alta que sugiere relevancia práctica en la variable analizada^22^ ^-^ ^23^.

Asimismo, se realizó un análisis de datos por protocolo, incluyendo a los participantes que terminaron la intervención; se observó un tamaño del efecto grande (d de Cohen=1,19 con intervalo de confianza del 95%) en la comparación entre el Grupo Experimental (Media=44,39, Desviación Estándar=7,54, tamaño de la muestra=46) y el Grupo Comparación (Media=35,38, Desviación Estándar=7,67, tamaño de la muestra=37). Este resultado indica que la intervención tuvo un efecto positivo.

Considerando el tamaño de la muestra del presente estudio, se realizó un ajuste calculando la *g* de Hedges, ya que proporciona una estimación insesgada del tamaño del efecto, especialmente adecuada para tamaños de muestra finitos. Se obtuvo un valor de *g*=1,18 [Intervalo de Confianza del 95% (0,71;1,64)], lo que indica un efecto grande de la intervención sobre la competencia para el cuidado.

### Satisfacción con la intervención de preparación para la transición del hospital al hogar

La mayor parte de los participantes del Grupo Experimental calificó la intervención Plan Cuidarte como excelente, destacando la calidad de la educación brindada para el cuidado de la IC, las herramientas utilizadas y la actitud del personal que la impartió (74%, 80% y 76% de los participantes calificaron estas características como excelentes, respectivamente). Respecto al Grupo Comparación, la mayoría de los participantes calificó el proceso de preparación para el egreso como bueno, destacando la necesidad de mejorar aspectos como el uso de herramientas para la educación.

## Discusión

Este estudio evaluó el efecto de la intervención de Enfermería Plan Cuidarte implementada durante la transición del hospital al hogar sobre la competencia para el cuidado de personas con IC, comparándola con la atención de egreso convencional. Las personas que recibieron el Plan Cuidarte presentaron mejores resultados que el Grupo Comparación (con diferencias estadísticamente significativas) en las dimensiones de la competencia para el cuidado relacionadas con el conocimiento, el instrumental y la anticipación, como así también en el nivel de competencia global para el cuidado.

La literatura indica que las intervenciones de Enfermería basadas en teorías producen mejores resultados que la atención convencional^24^. Para este estudio, la Teoría de las Transiciones permitió estructurar la intervención Plan Cuidarte, que superó al plan de egreso convencional. De igual forma, el marco de Whittemore^18^ facilitó el diseño y evaluación de esta intervención estructurada y con potencial de replicación para favorecer la práctica basada en la evidencia.

A partir de estos hallazgos y gracias a la educación del paciente como respaldo para mejorar y controlar su enfermedad, se espera que las personas que recibieron el Plan Cuidarte tengan una transición fluida del hospital al hogar dado que, tal como se ha documentado, lo anterior resulta crucial para mejorar los resultados en pacientes con IC^25^.

Estos hallazgos ratifican los de otros estudios a nivel nacional realizados en pacientes con enfermedades crónicas que han reportado que los planes de egreso estructurados mejoran dicha competencia^8^.

Este cambio positivo logrado en el nivel de competencia para el cuidado a partir del Plan Cuidarte responde a la necesidad de fortalecer a este grupo de pacientes, en quienes se ha reportado que, previo a la implementación de un programa de egreso para personas con enfermedad cardiovascular, la competencia para el cuidado no era óptima para manejar su condición de salud^8^.

La presente investigación también ratifica hallazgos sobre el perfil epidemiológico de los pacientes con IC reportado en investigaciones anteriores. Tanto en este caso como en los otros documentados, la mayoría de los pacientes diagnosticados con IC son adultos mayores y del sexo masculino^6^ ^,^ ^26^ ^-^ ^28^.

Los hallazgos reportados en la presente investigación sobre escolaridad, estado civil, estrato socioeconómico y presencia de un único cuidador también coinciden con estudios anteriores^5^.

Adicionalmente, los resultados evidencian una importante brecha en la alfabetización digital, especialmente en tecnologías que requieren habilidades técnicas avanzadas; esto resalta la necesidad de fortalecer la formación en el uso de computadores e Internet. Un estudio sobre la experiencia de pacientes con IC en el uso de las TIC para la autogestión reporta que esta baja alfabetización puede generar experiencias negativas, por lo que se recomienda brindar apoyo profesional al incorporar nuevas tecnologías en su cuidado^29^. Este apoyo debe preverse como un elemento significativo para incluir en el cuidado de la salud de estas personas, puesto que utilizar las TIC en la atención transicional de pacientes con IC puede mejorar su calidad de vida y su satisfacción con la atención, e incluso disminuir sus reingresos hospitalarios^30^.

Es importante señalar que en el curso de la presente investigación falleció el 3% de los pacientes hospitalizados. Esto coincide con lo que expresa el estudio *Recolfaca*, donde se reporta que el 3%-10% de los pacientes que son hospitalizados por IC fallecen dentro del hospital^5^. Esta elevada mortalidad vuelve a poner de manifiesto la necesidad de desarrollar intervenciones para prevenir y controlar las descompensaciones, así como para reducir la cantidad de reingresos hospitalarios en pacientes con IC^6^.

Respecto a las implicaciones de esta investigación para la práctica y para futuros estudios, es esencial considerar que la IC es la principal causa de hospitalización en personas mayores de 65 años^31^. Por ello, resulta pertinente evaluar el efecto del Plan Cuidarte en resultados clínicos como reingresos hospitalarios, visitas a servicios de urgencias y mortalidad. En consecuencia, se requiere un enfoque longitudinal para analizar el impacto de las intervenciones a lo largo del tiempo y en distintas poblaciones, especialmente en adultos mayores, quienes presentan mayor vulnerabilidad a complicaciones tras el alta^32^. Tal como se ha propuesto, este seguimiento podría incorporar herramientas de telesalud, telemonitoreo, seguimiento telefónico o visitas domiciliarias^6^, lo que implicaría integrar la capacitación digital como parte del proceso de cuidado.

Del mismo modo, en estudios posteriores será importante analizar la efectividad del Plan Cuidarte incluyendo a los cuidadores de pacientes con IC, dado que las intervenciones dirigidas al egreso han demostrado mejorar la competencia para el cuidado de la diada paciente/cuidador^8^.

Los resultados del presente estudio resaltan la importancia de continuar desarrollando e implementando programas de intervenciones de Enfermería estructurados y personalizados que mejoren la competencia para el cuidado de los pacientes con IC. Estos programas deben integrarse en el proceso de alta hospitalaria y garantizar una transición fluida hacia la atención ambulatoria de manera más frecuente y continua y liderada por profesionales de Enfermería^33^.

Asimismo, mejorar la competencia para el cuidado en el paciente es crucial a fin de manejar su enfermedad, buscando los mejores resultados clínicos posibles y una experiencia positiva en su manejo^34^. El Plan Cuidarte puede ser útil, riguroso y completo para pacientes con IC que deben continuar sus cuidados en el hogar.

A pesar de los hallazgos obtenidos en esta investigación, es necesario señalar que la competencia para el cuidado reportada por los pacientes con IC corresponde a un dato autoinformado y que se evaluó en un periodo de seguimiento corto. Esto limita la posibilidad de determinar sus implicaciones prácticas y su relación costo-beneficio en desenlaces clínicos y posteriores al egreso, como calidad de vida, estado general de salud, aparición de complicaciones, reingresos, duración de la estancia hospitalaria o índices de mortalidad, entre otros. De igual forma, es preciso indicar que estos hallazgos hacen referencia a un grupo específico de pacientes y que el estudio debe ser replicado para poder generalizarlos a otras poblaciones o contextos.

## Conclusión

El Plan Cuidarte es una intervención sistematizada y teóricamente fundamentada que, al aplicarse durante la estancia hospitalaria, mejora de manera significativa la competencia para el cuidado de las personas con IC durante el periodo de transición del hospital al hogar.

Su estructura metodológica y su base en marcos conceptuales propios de la disciplina respaldan su replicabilidad y pertinencia para integrarse a la práctica clínica habitual. Los resultados demuestran que la participación activa de los profesionales de Enfermería es esencial para favorecer una transición más segura y contribuir a una mejor gestión de la enfermedad y a resultados de salud más favorables. De este modo, el estudio reafirma el valor estratégico de las intervenciones de Enfermería durante el egreso hospitalario y aporta evidencias que fortalecen prácticas efectivas y sostenibles en el cuidado de personas con enfermedades crónicas como la IC.

## Data Availability

El conjunto de datos de este artículo está disponible en el enlace https://doi.org/10.6084/m9.figshare.29361920.v1
